# Epigenetic alteration of smooth muscle cells regulates endothelin-dependent blood pressure and hypertensive arterial remodeling

**DOI:** 10.1172/JCI186146

**Published:** 2025-03-27

**Authors:** Kevin D. Mangum, Qinmengge Li, Katherine Hartmann, Tyler M. Bauer, Sonya J. Wolf, James Shadiow, Jadie Y. Moon, Emily C. Barrett, Amrita D. Joshi, Gabriela Saldana de Jimenez, Zara Ahmed, Rachael Wasikowski, Kylie Boyer, Andrea T. Obi, Frank M. Davis, Lin Chang, Lam C. Tsoi, Johann Gudjonsson, Scott M. Damrauer, Katherine A. Gallagher

**Affiliations:** 1 Section of Vascular Surgery, Department of Surgery,; 2 Department of Microbiology and Immunology, and; 3 Department of Dermatology University of Michigan, Ann Arbor, Michigan, USA.; 4 Department of Surgery, University of Pennsylvania, Philadelphia, Pennsylvania, USA.; 5 Department of Internal Medicine, University of Michigan, Ann Arbor, Michigan, USA.

**Keywords:** Cardiology, Genetics, Epigenetics, Hypertension, Transcription

## Abstract

Long-standing hypertension (HTN) affects multiple organs and leads to pathologic arterial remodeling, which is driven by smooth muscle cell (SMC) plasticity. To identify relevant genes regulating SMC function in HTN, we considered Genome Wide Association Studies (GWAS) of blood pressure, focusing on genes encoding epigenetic enzymes, which control SMC fate in cardiovascular disease. Using statistical fine mapping of the KDM6 Jumonji domain-containing protein D3 (*JMJD3*) locus, we found that rs62059712 is the most likely casual variant, with each major T allele copy associated with a 0.47 mmHg increase in systolic blood pressure. We show that the T allele decreased *JMJD3* transcription in SMCs via decreased SP1 binding to the *JMJD3* promoter. Using our unique SMC-specific Jmjd3-deficient murine model (*Jmjd3^fl/fl^Myh11^CreERT^*), we show that loss of *Jmjd3* in SMCs results in HTN due to decreased endothelin receptor B (*EDNRB*) expression and increased endothelin receptor A (*EDNRA*) expression. Importantly, the EDNRA antagonist BQ-123 reversed HTN after Jmjd3 deletion in vivo. Additionally, single-cell RNA-Seq (scRNA-Seq) of human arteries revealed a strong correlation between *JMJD3* and *EDNRB* in SMCs. Further, JMJD3 is required for SMC-specific gene expression, and loss of JMJD3 in SMCs increased HTN-induced arterial remodeling. Our findings link a HTN-associated human DNA variant with regulation of SMC plasticity, revealing targets that may be used in personalized management of HTN.

## Abstract 2

The histone demethylase, JMJD3, in vascular smooth muscle cells regulates blood pressure. JMJD3 is required for expression of endothelin receptor A and smooth muscle genes.

## Graphical abstract

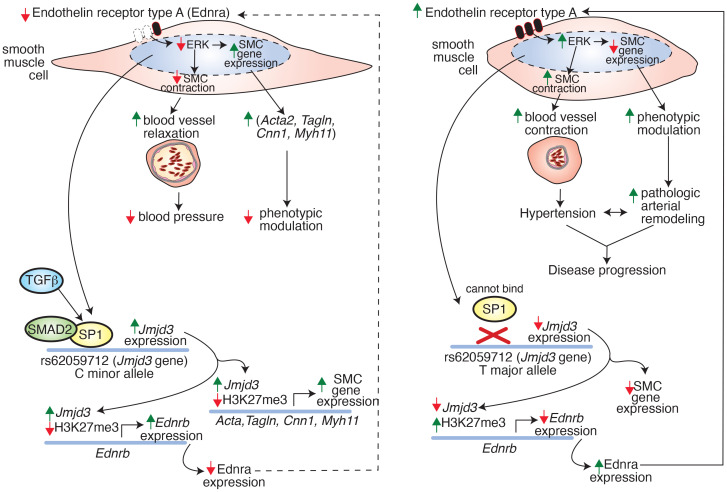

## Introduction

Hypertension (HTN) contributes to substantial morbidity and mortality in the United States due to its detrimental effects on end organs including the cardiovascular system (1). Blood pressure (BP) is directly regulated by peripheral vascular resistance (vessel tone), which is mediated by vascular smooth muscle cells (SMCs) (2). Proper BP control requires vascular SMC contraction and relaxation, which is regulated by pharmacologic and mechanical stimuli. Endothelin-1 (ET-1), a potent vasoconstrictor, binds to the endothelin receptor, leading to myosin light chain phosphorylation and SMC contraction. Two endothelin receptors are expressed in SMCs (A and B); however, endothelin receptor A (EDNRA) is the predominant receptor mediating SMC contractility and vessel tone (3). In contrast, the effect of endothelin receptor B (EDNRB) on SMC function is less established, since recent reports have indicated dual roles for EDNRB (3–5). Endothelin receptor activation mediates SMC contractility and HTN, and endothelin receptor blockade decreases BP in experimental models of HTN (4).

SMC contractility/function is determined by SMC phenotype, which alternates between contractile or synthetic phenotypes, depending on upstream cues. The contractile phenotype is induced by mechanical (e.g., stretch) and molecular (e.g., TGF-β) stimuli, which modulate downstream gene expression via the transcription factors (TFs), serum response factor (SRF), myocardin, and the myocardin-related transcription factors (MRTFs) (6–9). Other TFs such as SP1 and the SMAD family induce genes required for the contractile SMC phenotype (10, 11). In contrast, the synthetic SMC phenotype occurs in atherosclerosis, HTN, and restenosis and is driven by PDGF, inflammatory cytokines, and bone morphogenic protein (12–14). The TFs KLF4 and ELK1 repress SMC differentiation and promote the synthetic phenotype (15, 16). The precise regulation of SMC differentiation by these upstream cues is highly relevant in cardiovascular disease during which vascular SMCs become phenotypically modulated and contribute to pathology. Specifically, during disease, SMC genes (*ACTA2*, *TAGLN*, *CNN1*, *MYH11*) are repressed while proliferative-associated genes (*KLF4*, *FOS*) are increased (13, 17). Epigenetic mechanisms have been previously shown by our group and others to regulate cell phenotypes and control downstream gene expression in homeostatic and pathologic states (18–20). In particular, epigenetic alteration of chromatin structure by epigenetic enzymes and TFs influences SMC phenotype during development and cardiovascular pathology (21–27).

Genome wide association studies (GWAS) have identified numerous genetic loci associated with cardiovascular diseases including HTN (28). Several GWAS for BP have identified a genome-wide significant association for systolic BP (SBP) at the Jumonji domain-containing protein D3 (*JMJD3*) (also known as *KDM6B*) locus, which encodes the histone demethylase JMJD3 (29). Notably, JMJD3 is known to play a role in abdominal aortic aneurysm formation and control inflammatory gene expression in innate immune cells in cardiometabolic disease (30, 31).

Here, we use statistical fine mapping to prioritize rs62059712 as the single high-confidence causal signal at the *JMJD3* locus. We use mechanistic studies to link this genetic variant to SBP through changes in *JMJD3* transcription in human and murine SMCs via SP1 TF binding. SMC-specific deletion of *Jmjd3* in mice (*Jmjd3^fl/fl^Myh11^CreERT^*) resulted in increased BP in an angiotensin II (Ang II) model of HTN, which was due to decreased *EDNRB* and increased EDNRA, genes encoding expression of EDNRA and EDNRB, respectively, in SMCs. Endothelin receptor antagonism eliminated increased vessel contractility after JMJD3 deletion ex vivo and in vivo. Additionally, JMJD3 loss in SMCs enhanced endothelin-ERK activation, resulting in increased SMC phenotypic modulation after vascular injury (i.e., chronic HTN). Our study provides a mechanistic link among human genetic variation, *JMJD3* expression in SMCs associated with BP alterations, and an SMC phenotypic switch in a murine model and also identifies a genetic target that may be used to develop previously unknown anti-HTN therapies.

## Results

### rs62059712 minor C allele increases JMJD3 transcription via enhanced SP1 binding to a regulatory region in the JMJD3 promoter.

During cardiovascular diseases (e.g., atherosclerosis), SMCs lose their mature, contractile markers, and switch to a synthetic, proliferative phenotype (13). There remains a knowledge gap of the specific downstream mechanisms that underlie SMC plasticity. Our group and others have identified that epigenetic pathways regulate cell plasticity in cardiovascular disease (27, 32). To identify translationally relevant epigenetic mechanisms regulating BP, we analyzed large GWAS for BP to identify human variants located within or near genes encoding various chromatin-modifying enzymes (CMEs) with previously identified roles in cardiovascular disease (29, 33, 34). Our most promising target was the genome-wide association between the *KDM6B* (*JMJD3)* locus and SBP. *JMJD3* encodes a histone demethylase that regulates macrophage phenotype in cardiometabolic diseases (30, 31, 35). The major allele (T) of the lead variant rs62059712 is associated with increased SBP (0.47 mmHg increase; CI 0.28–0.56). A phenome-wide association analysis in OpenGWAS and the GWAS Catalog identified association of rs62059712 with other related phenotypes, including diastolic BP (DBP), pulse pressure, and use of antihypertensive agents including those acting on the renin-angiotensin system, calcium channel blockers, and diuretics (Supplemental Table 1; supplemental material available online with this article; https://doi.org/10.1172/JCI186146DS1).

To refine the signal at the *JMJD3* locus and prioritize potential causal variants, we performed statistical fine mapping using Bayes factor analysis. We considered a 500 kb locus, 250 kb upstream and downstream of rs62059712. Our analysis identified the lead variant, rs62059712, as the single high-confidence causal signal at this locus (posterior inclusion probability of 0.98, Table 1), providing strong supportive evidence for this variant. In addition to the candidate variant, we also considered the effect of SNPs in high linkage disequilibrium (LD). Using HaploReg (https://pubs.broadinstitute.org/mammals/haploreg/haploreg_v4.1.php) we identified an additional variant, rs74480102, approximately 2.4 KB downstream of rs62059712 in strong LD with rs62059712 (defined in this study as *r^2^* > 0.3 and D’ = 1, where D’ is the normalized disequilibrium coefficient).

**Figure 1:**
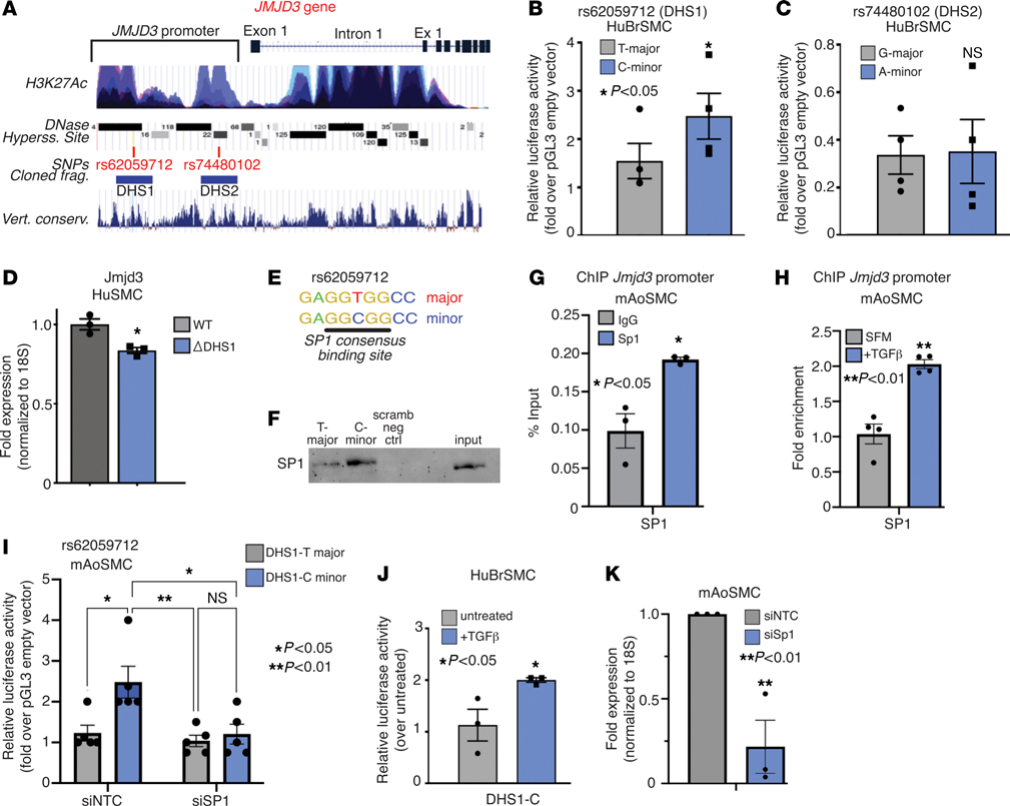
rs62059712 minor C allele increases JMJD3 transcription via enhanced SP1 binding. (**A**) Schematic of the human *JMJD3* gene, including gene structure, H3K27Ac, DHS, BP-associated SNPs, cloned fragments for luciferase assays, and vertebrate conservation (UCSC genome browser). (**B**) Luciferase results of DHS1 rs62059712 (T-major vs. C-minor) and (**C**) DHS2 rs74480102 (G-major vs. A-minor) in cultured primary human bronchial smooth muscle cells (HuBrSMCs). (**D**) qPCR for *JMJD3* in HuSMCs with CRISPR/Cas9-mediated deletion of a 450 bp region encompassing rs62059712 within DHS1 (ΔDHS1) compared with unedited (WT) HuSMCs. (**E**) Sequence of rs62059712 T-major and C-minor sequences with SP1 consensus binding site underlined. (**F**) Western blot for SP1 after affinity purification using T versus C probes corresponding to rs62059712 SNP region incubated with human aortic smooth muscle cell (HuAoSMC) nuclear lysate. (**G**) ChIP-qPCR for Sp1 at the *Jmjd3* promoter in mAoSMCs compared with IgG negative control. (**H**) ChIP-qPCR for Sp1 at the *Jmjd3* promoter in mAoSMCs serum starved or treated with TGF-β. (**I**) Luciferase assays in mAoSMCs treated with siNTC and siSp1 siRNAs, then transfected with pGL3-DHS1-T and -C. (**J**) Luciferase assays in HuSMCs transfected with DHS1-C construct and then treated with TGF-β (20 ng/ml). (**K**) qPCR for *Jmjd3* after NTC versus Sp1 knockdown in mAoSMCs serum starved for 16 hours. Data are represented as mean ± SEM. Results are representative of data from SMCs from 4–6 mice per group. *n* = 3 independent experiments. Two-tailed Student’s *t* test, *P < 0.05; **P < 0.01.

To locate rs62059712 and rs74480102 in relation to the *JMJD3* gene and possible alignment with features of open chromatin, we utilized the publicly accessible UCSC Genome Browser (36). Notably, rs62059712 and rs74480102 were both located upstream of the *JMJD3* gene within regions that aligned with several features of active transcription including H3K27 acetylation ChIP-Seq peaks, DNase hypersensitive sites (DHS), and vertebral conservation (Figure 1A). Thus, we hypothesized that rs62059712 (within DHS1) and/or rs74480102 (within DHS2) altered *JMJD3* transcription by influencing chromatin and TF accessibility to one or both regions. Since SMC contractility directly regulates vascular tone, and thus BP, and because our phenome-wide analysis linked the rs62059712 genotype to antihypertensive agents that act on SMCs, we hypothesized that *JMJD3* expression in SMCs was important for BP regulation. To test this, we generated an allelic series of luciferase reporter constructs corresponding to major and minor alleles for both SNPs, transfected each into human SMCs (HuSMCs), and measured luciferase activity. The rs62059712 minor C allele demonstrated increased transcriptional activity compared with the major T allele in HuSMCs and mouse aortic SMCs (mAoSMCs) (Figure 1, B and C, and Supplemental Figure 1, A and B). Notably, there was no difference in luciferase activity between the rs74480102 major G and minor A alleles (Figure 1C). Importantly, rs62059712 did not exhibit allele-specific differences in activity in other vascular cells, including bone marrow–derived macrophages (BMDMs) or endothelial cells (ECs) (Supplemental Figure 1, C and D). Next, to test the effect of the rs62059712 variant on *JMJD3* expression, we used CRISPR/Cas9 to delete the 450 bp region encompassing the SNP in HuSMCs. Deletion of this region resulted in decreased *JMJD3* expression, further supporting the role of rs62059712 in regulating *JMJD3* expression (Figure 1D).

To define the mechanism whereby the rs62059712 minor C allele resulted in increased transcriptional activity, we analyzed the region of the *JMJD3* gene containing the rs62059712 C allele sequence for predicted TF binding sites using the JASPAR web-based tool (37). We found the C-containing sequence conformed to a predicted SP1-binding site (Figure 1E). Additionally, conservation analysis revealed that the region containing the SP1-binding site was conserved across rodent species, although the major T allele–containing sequence was conserved (Table 2). We performed affinity purification of TFs in HuSMC nuclear lysates using biotin-tagged DNA oligonucleotides that corresponded to rs62059712 minor C, major T, or scrambled negative control sequences. The minor C allele bound the TF, SP1, with greater affinity than the major T allele (Figure 1F). Because SP1 can interact with the TF SMAD2 to drive TGF-β–dependent *ACTA2* expression and because the SMAD family of TFs are known regulators of TGF-β–dependent SMC differentiation, we determined whether SMAD2 immunoprecipitated with the C-minor allele probe (10). We found increased SMAD2 binding to the C-containing sequence compared with the T-containing sequence (Supplemental Figure 1E). Next, we used ChIP in mAoSMCs to demonstrate Sp1 binding at the *Jmjd3* promoter in vivo (Figure 1G). Furthermore, given that TGF-β is a known driver of SMC differentiation, we assessed the effect of TGF-β treatment on Sp1 binding to the *Jmjd3* promoter (11, 38). As demonstrated in Figure 1H, Sp1 binding to the murine *Jmjd3* promoter was increased in ChIP experiments following TGF-β stimulation of mAoSMCs. To determine whether Sp1 mediated the increased transcription activity of the minor C allele, we transfected DHS1-T and -C minor allele luciferase constructs in mAoSMCs treated with siRNA against *Sp1* or a nontargeting control (NTC) siRNA. Interestingly, Sp1 knockdown in mAoSMCs abolished the increased transcriptional activity of the C minor allele, decreasing it to that of the T major allele (Figure 1I). Additionally, TGF-β stimulation of mAoSMCs resulted in a 2-fold increase in luciferase activity of the DHS1-C fragment (Figure 1J). Since the DHS1 region was required for *JMJD3* transcription (see Figure 1D) and SP1 binding to this region mediated its allele-specific activity in SMCs, we hypothesized that SP1 was required for *JMJD3* expression. We performed siRNA knockdown of Sp1 in mAoSMCs and measured *Jmjd3* expression. Sp1 knockdown significantly decreased *Jmjd3* expression in mAoSMCs (Figure 1K). Additionally, because SMAD2 also bound the *JMJD3* minor C allele, perhaps via its interaction with SP1, we tested to determine whether SMAD2 regulated *JMJD3* expression. siRNA knockdown of Smad2 decreased *Jmjd3* expression in mAoSMCs (Supplemental Figure 1F). Taken together, these results demonstrate that the BP-associated human variant rs62059712 minor C allele increases *JMJD3* transcription in SMCs via increased SP1 binding.

### JMJD3 loss in vascular SMCs results in HTN.

**Figure 2:**
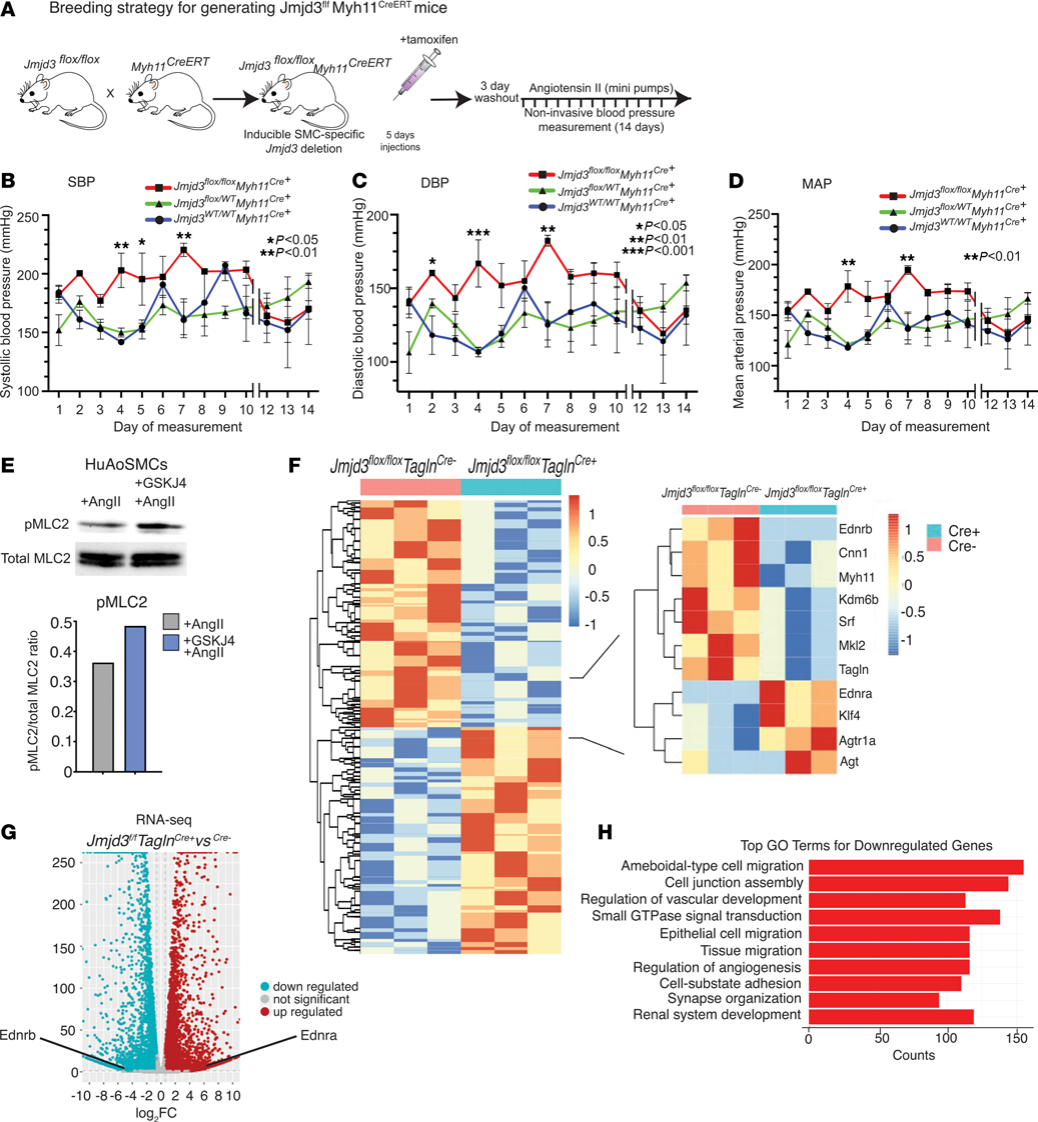
JMJD3 loss in vascular SMCs results in HTN. (**A**) Schematic depicting experiment and breeding strategy for generating inducible SMC-specific Jmjd3-knockout *Jmjd3^fl/fl^Myh11^CreERT^* mice. BP obtained by tail cuff for 14 days in WT, heterozygous, and homozygous *Jmjd3^fl/fl^Myh11^CreERT^* mice treated with Ang II via osmotic minipumps. (**B**) SBP, (**C**) DBP, and (**D**) MAP are depicted. *n* = 4–6 mice per genotype/group. (**E**) HuAoSMCs were treated with Ang II (100 nM) or Ang II and GSKJ4 (50 nM) and then analyzed for pMLC2 and total MLC2 by Western blotting. Blot is representative of *n* = 3 independent experiments with representative densitometry depicted below. (**F**) DEGs obtained from RNA-Seq analysis of cultured mAoSMCs isolated from *Jmjd3^fl/fl^Tagln^Cre+^* and *Jmjd3^fl/fl^Tagln^Cre–^* mice with relevant DEGs depicted to right. DEGs depicted met significant threshold of *P* < 0.05. Results obtained are representative of samples for each genotype submitted in triplicate and obtained from *n* = 6–8 mice per sample. (**G**) Volcano plot for upregulated (red) and downregulated (blue) DEGs with fold-change expression depicted on *x* axis. Locations of *Ednrb* and *Ednra* are annotated. (**H**) Bar graph of GO analysis for top 10 downregulated genes in *Jmjd3^fl/fl^Tagln^Cre+^* SMCs from RNA-Seq results. Gene pathways are listed on *y* axis and number of gene counts for each pathway are listed on *x* axis. Data are represented as mean ± SEM. *n* = 3 independent experiments for in vitro studies. Two-way ANOVA. *P < 0.05; **P < 0.01; ***P < 0.001.

Given our identification of a BP-associated gene regulatory region within the human *JMJD3* promoter that displayed allele-specific activity in vascular SMCs, we hypothesized that JMJD3 was required for SMC-mediated vasomotor tone and BP. To determine the role of JMJD3 in SMCs in BP, we created a SMC-specific Jmjd3 deletion murine model by crossing our *Jmjd3^fl/fl^* mice (along with *Jmjd3^WT/WT^* and *Jmjd3^fl/WT^* mice) with tamoxifen-inducible *Myh11^CreERT^* mice (to generate *Jmjd3^fl/fl^Myh11^CreERT^* mice) (Figure 2A). To eliminate the effect of tamoxifen on BP and test the roles of heterozygous and homozygous *Jmjd3* deletion, we treated all *Jmjd3^WT/WT^Myh11^CreERT^*, *Jmjd3^fl/WT^Myh11^CreERT^*, and *Jmjd3^fl/fl^Myh11^CreERT^* littermates with tamoxifen for 5 days, followed by a 3-day “washout” period, to generate WT, *Jmjd3^fl/WT^Myh11^Cre+^*, and *Jmjd3^fl/fl^Myh11^Cre+^* mice, respectively. We then implanted osmotic minipumps (ALZET, Model 2004) filled with saline or Ang II and measured BP for 14 days in response to saline or Ang II infusion (1 μg/kg/min). SMC-specific deletion of *Jmjd3* (in *Jmjd3^fl/fl^Myh11^Cre+^* mice) resulted in significantly higher SBP, DBP, and mean arterial pressure (MAP) in response to Ang II compared with heterozygote and WT controls (Figure 2, B–D). *Jmjd3^fl/fl^Myh11^Cre+^* mice had significantly increased 14-day averaged SBP, DBP, and MAP compared with littermate controls (Supplemental Figure 2, A–C). Because BP is regulated by non-SMC vascular cells (e.g., ECs), we generated endothelial- (*Jmjd3^fl/fl^Cdh5^CreERT^*) and myeloid-specific (*Jmjd3^fl/fl^Lyz2^Cre^*) murine models of Jmjd3 deletion and measured their BPs. Importantly, Jmjd3 deletion in ECs or myeloid cells did not significantly affect BP (Supplemental Figure 2, D–I).

Since arterial SMCs are the main cell type regulating vascular tone/BP, we hypothesized that JMDJ3 deletion resulted in increased vascular SMC contractility, thereby leading to increased arterial tone/BP. SMC contraction occurs after activation of calcium/calmodulin-dependent phosphorylation of myosin light chain kinase, resulting in myosin light chain 2 (pMLC2) phosphorylation (3). Thus, as an index of SMC contractility, we measured pMLC2 via Western blotting and found it was increased in human aortic SMCs (HuAoSMCs) treated with a JMJD3-specific inhibitor (GSKJ4, 50 nM) plus Ang II (100 nM) compared with Ang II only (control) (Figure 2E). To elucidate the mechanism of JMJD3-dependent SMC contractility and BP, we performed RNA-Seq on cultured aortic SMCs isolated from *Jmjd3^fl/fl^Tagln^Cre^* mice. *Jmjd3* mRNA levels in *Jmjd3^fl/fl^Tagln^Cre+^* SMCs were nearly 100% depleted (Supplemental Figure 3A). Interestingly, among the many genes differentially regulated, we identified several canonical SMC-specific genes (*TAGLN*, *CNN1*, *MYH11*, *SRF*, *MKL2*) that were strongly downregulated in *Jmjd3^fl/fl^Tagln^Cre+^* SMCs (Figure 2F). SMC-specific marker proteins in aortic tissue from *Jmjd3^fl/fl^Tagln^Cre+^* mice were also decreased (Supplemental Figure 3B). In our RNA-Seq data, *Klf4*, a TF that represses SMC differentiation, was upregulated in *Jmjd3^fl/fl^Tagln^Cre+^* SMCs compared with *Jmjd3^fl/fl^Tagln^Cre–^* SMCs (see Figure 2F). Given the importance of endothelin signaling in SMCs in BP regulation, we examined endothelin receptor expression in our RNA-Seq data and identified a 6-fold downregulation in *Ednrb* expression and 6-fold upregulation in *Ednra* in *Jmjd3^fl/fl^Tagln^Cre+^* SMCs (Figure 2G). Other genes associated with HTN including *AGTR1A* and *AGT* were increased in *Jmjd3^fl/fl^Tagln^Cre+^* SMCs (see Figure 2F). Gene Ontology (GO) analysis of our RNA-Seq results identified common pathways of migration, vascular development, angiogenesis, and signal transduction that were downregulated in *Jmjd3^fl/fl^Tagln^Cre+^* SMCs compared with control (Figure 2H). Complementary GO analysis of upregulated genes in *Jmjd3^fl/fl^Tagln^Cre+^* SMCs revealed pathways common to DNA and RNA processes (Supplemental Figure 3C). Taken together, these data show that JMJD3 in SMCs regulates contractility and BP and controls expression of genes associated with SMC phenotype and HTN.

### JMJD3 is required for EDNRB expression in SMCs and suppresses the hypertensive gene program.

**Figure 3:**
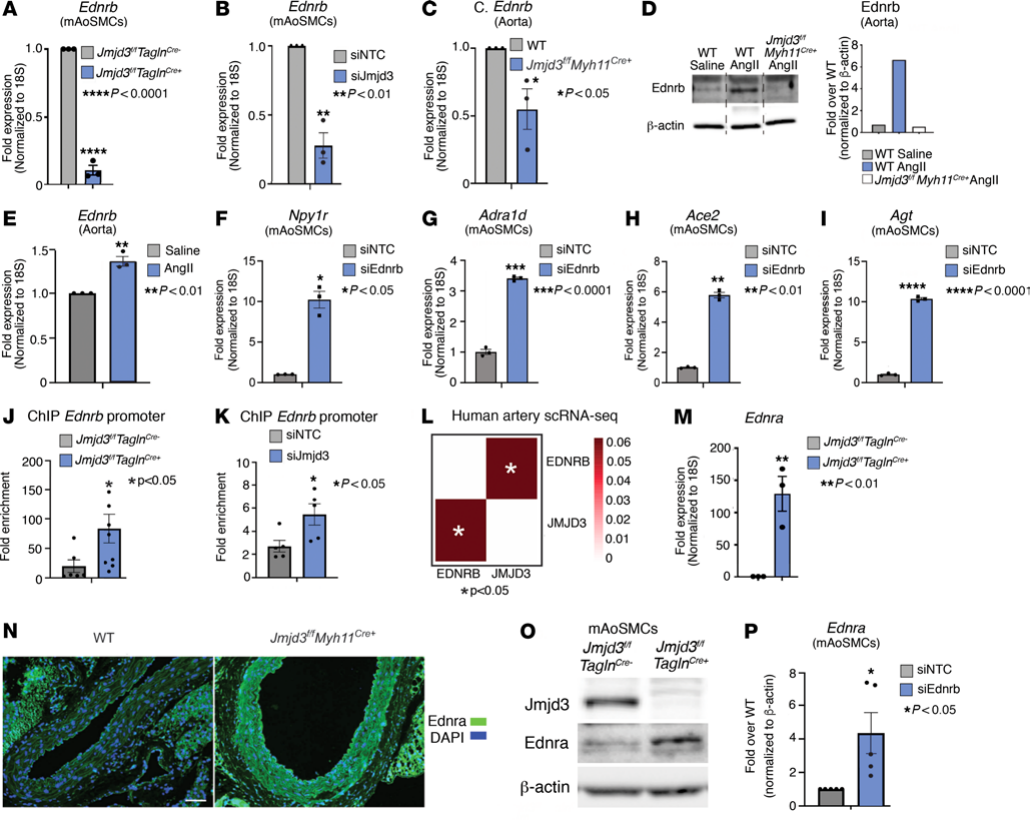
JMJD3 is required for *EDNRB* expression in SMCs and suppresses the hypertensive gene program. (**A**) qPCR of *Ednrb* in SMCs isolated from *Jmjd3^fl/fl^Tagln^Cre+^* and *Jmjd3^fl/fl^Tagln^Cre–^* mice. (**B**) qPCR for *Ednrb* in mAoSMCs treated with siNTC or siJmjd3 siRNA and then serum starved for 16 hours. (**C**) qPCR of *Ednrb* in whole aortic tissue from *Jmjd3^fl/fl^Myh11^Cre+^* and WT mice. (**D**) Representative Western blot and densitometry results for Ednrb in aortas isolated from *Jmjd3^fl/fl^Myh11^Cre+^* and WT mice after 14 days of Ang II or saline infusion. Representative densitometry depicted to right. (**E**) qPCR for *Ednrb* in aortas isolated from WT mice treated with saline or Ang II for 14 days. (**F**) qPCR for *Npy1r* (**F**), *Adra1d* (**G**), *Ace2* (**H**), and *Agt* (**I**) after Ednrb knockdown in mAoSMCs, then serum starved for 16 hours. (**J**) ChIP-qPCR for H3K27me3 at *Ednrb* promoter in *Jmjd3^fl/fl^Tagln^Cre^* mAoSMCs. (**K**) ChIP-qPCR for H3K27me3 at the *Ednrb* promoter in mAoSMCs treated with siNTC or siJmjd3 siRNA. (**L**) scRNA-Seq data from human femoral arteries showing Pearson’s correlation between *JMJD3* and *EDNRB* in SMCs. *n* = 4 samples. (**M**) qPCR of *Ednra* in SMCs isolated from *Jmjd3^fl/fl^Tagln^Cre^* mice. (**N**) Representative images of immunofluorescent staining for Ednra in aortas harvested from *Jmjd3^fl/fl^Myh11^Cre+^* and WT mice in (**D**). Scale bar: 50 μm. (**O**) Representative Western blotting results for Jmjd3 and Ednra in mAoSMCs from *Jmjd3^fl/fl^Tagln^Cre+^* or *Jmjd3^fl/fl^Tagln^Cre–^* mice. (**P**) qPCR for *Ednra* in mAoSMCs treated with siNTC or siEdnrb siRNA, then serum starved for 16 hours. Data are represented as mean ± SEM. *n* = 3 independent experiments, representative of 4–6 mice per group. Two-tailed Student’s *t* test. *P < 0.05; **P < 0.01; *** *P* < 0.0001.

To further analyze the downstream transcription targets of JMJD3 that drive BP regulation in vascular SMCs, we performed a superarray of well-established genes involved in HTN in aortic SMCs isolated from *Jmjd3^fl/fl^Tagln^Cre^* mice. In alignment with our RNA-Seq data, there was a 150-fold reduction in expression of *Ednrb* in *Jmjd3^fl/fl^Tagln^Cre+^* SMCs and upregulation of genes known to increase BP (e.g., *Ednra*, *Agt*, *Ace2*) (Supplemental Figure 4A). Superarray results were confirmed using quantitative PCR (qPCR), which demonstrated decreased *Ednrb* expression (and increased expression of HTN genes) in *Jmjd3^fl/fl^Tagln^Cre+^* SMCs (Figure 3A and Supplemental Figure 4, B–H). Further, siRNA knockdown of Jmjd3 in mAoSMCs led to increased HTN gene expression, including *Edn1* (Supplemental Figure 4, I and J). To further examine the regulation of *EDNRB* by JMJD3, we performed siRNA knockdown of Jmjd3 in mAoSMCs and found reduced *Ednrb* expression compared with a NTC siRNA (Figure 3B). Notably, because SP1 was required for *JMJD3* transcription, we tested to determine whether SP1 played a similar role in regulating endothelin receptor expression. Interestingly, siRNA knockdown of Sp1 in mAoSMCs resulted in increased *Ednra* expression (Supplemental Figure 5). To examine regulation of *EDNRB* by JMJD3 in vivo, we harvested aortas from tamoxifen-injected *Jmjd3^fl/fl^Myh11^CreERT^* (*Jmjd3^fl/fl^Myh11^Cre+^*) and *Jmjd3^WT/WT^Myh11^CreERT^* (WT) mice after 14-day treatment with Ang II (as described above in tail-cuff BP experiments) and found reduced *Ednrb* expression in aortas from mice with SMC-specific *Jmjd3* deletion (Figure 3C). Notably, increased Ednrb protein in aortic tissue in response to Ang II was abolished by SMC-specific *Jmjd3* deletion (Figure 3D). *Ednrb* in aortas was also increased at the mRNA level by Ang II treatment (Figure 3E). We tested the effects of EDNRB loss on downstream endothelin and HTN signaling using siRNA-mediated knockdown of Ednrb in mAoSMCs, which led to increased HTN gene expression (Figure 3, F–I). To determine whether the effects on *Ednrb* expression after Jmjd3 deletion were due to changes in H3K27me3, the epigenetic mark associated with Jmjd3, we performed ChIP in mAoSMCs from *Jmjd3^fl/fl^Tagln^Cre^* mice and observed increased H3K27me3 at the *Ednrb* promoter in Jmjd3-deficient SMCs (Figure 3J). Additionally, H3K27me3 enrichment was increased at the *Ednrb* promoter in mAoSMCs treated with *Jmjd3* siRNA compared with NTC siRNA (Figure 3K). Next, to determine whether the relationship between *JMJD3* and *EDNRB* expression was conserved in HuSMCs, we isolated femoral arteries from 4 HTN patients. Single-cell RNA-Seq (scRNA-Seq) and Pearson correlation analysis identified significant correlation between *JMJD3* and *EDNRB* in SMCs (Figure 3L). Since there are 2 endothelin receptors (A and B) and prior reports have identified dual roles for these receptors, we determined whether *EDNRA* expression levels were altered relative to *JMJD3* (3). In both our superarray (see Supplemental Figure 4A) and targeted qPCR in *Jmjd3^fl/fl^Tagln^Cre^* SMCs, we found increased *Ednra* expression in mAoSMCs after *Jmjd3* depletion (Figure 3M). We also observed increased Ednra by immunofluorescent staining of *Jmjd3^fl/fl^Myh11^Cre+^* aortas compared with aortas from WT mice (Figure 3N). Additionally, Ednra protein was increased in *Jmjd3^fl/fl^Tagln^Cre+^* SMCs (Figure 3O). Next, we performed siRNA knockdown of *Ednrb* in mAoSMCs and measured *Ednra* expression by qPCR. Interestingly, Ednrb knockdown led to a 4-fold increase in *Ednra* expression in mAoSMCs, suggesting an intricate transcriptional balance between *EDNRA* and *EDNRB* that is controlled by JMJD3 (Figure 3P). In sum, these results show JMJD3, likely via EDNRB, regulates SMC contractility and BP.

### JMJD3 regulates vessel tone via endothelin-ERK signaling in vascular SMCs.

**Figure 4:**
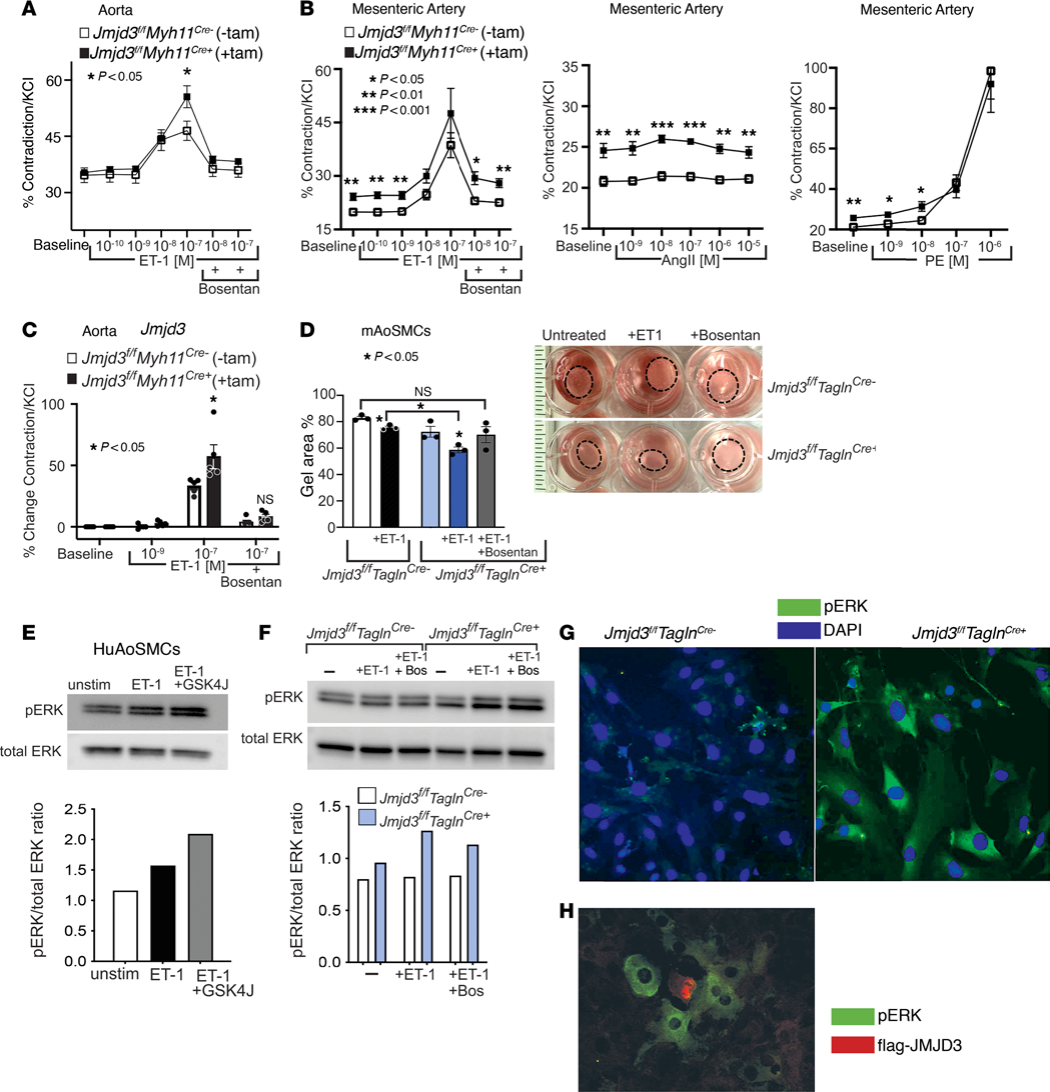
JMJD3 regulates vessel tone via endothelin-ERK signaling in vascular SMCs. (**A**) Contractility of aortas harvested from *Jmjd3^fl/fl^Myh11^Cre+^* and *Jmjd3^fl/fl^Myh11^Cre–^* mice in response to ET-1 (0–10^–7^ M) and bosentan (10^–8^–10^–7^ M). (**B**) Contractility of mesenteric artery segments harvested from same mice as in **A** in response to increasing concentrations of ET-1 (0–10^–7^ M) and bosentan (10^–8^–10^–7^ M), Ang II (0–10^–5^ M), or PE (0-10^–6^ M). (**C**) Graph depicting percentage change contractility of aortic rings treated with ET-1 (0–10^–7^ M) plus or minus bosentan (10^–7^ M). (**D**) Quantified gel areas containing cultured mAoSMCs from *Jmjd3^fl/fl^Tagln^Cre^* mice. Gels treated with ET-1 (1 μM), ET-1 plus bosentan (10 μM), or bosentan alone. Results depicted as percentage of initial gel area at 24 hours after seeding. (**E**) Representative Western blot of HuAoSMCs unstimulated, treated with ET-1 (1 μM), or ET-1 plus GSKJ4 (50 nM) and then probed for pERK (top) or total ERK (bottom). Blot with representative densitometry results underneath. (**F**) Representative Western blot of *Jmjd3^fl/fl^Tagln^Cre+^* and *Jmjd3^fl/fl^Tagln^Cre–^* control mAoSMCs unstimulated, treated with ET-1 (1 μM), or ET-1 plus bosentan (10 μm) and then probed for pERK (top) or total ERK (bottom). Blot with representative densitometry results underneath. (**G**) Representative immunofluorescent staining for pERK in *Jmjd3^fl/fl^Tagln^Cre+^* (right) and *Jmjd3^fl/fl^Tagln^Cre–^* (left) mAoSMCs (up to 4 high powered fields counted per experiment). (**H**) Representative immunofluorescent staining of endogenous pERK in mAoSMCs transfected with Flag-Jmjd3. Original magnification, ×20 (**G** and **H**). Data are represented as mean ± SEM. *n* = 3 independent experiments; 6 mice were included in each group for contractility experiments. In vitro experiments representative of SMCs from 4–6 mice per group. Two-way ANOVA (**A**–**C**) and 2-tailed Student’s *t* test (**D**). *P < 0.05; **P < 0.01; ***P < 0.001.

Since BP control is complex and regulated by multiple organ systems, we tested the effects of JMJD3 deletion on vessel tone independent of organ tissues. We isolated aortic and mesenteric artery segments from *Jmjd3^fl/fl^Myh11^CreERT^* mice injected with tamoxifen (*Jmjd3^fl/fl^Myh11^Cre+^*) or corn oil (*Jmjd3^fl/fl^Myh11^Cre–^*) and measured their contractility using a vessel ring assay in response to various contractile agonists, including ET-1, Ang II, and phenylephrine (PE) ex vivo. We observed that aortas isolated from *Jmjd3^fl/fl^Myh11^Cre+^* mice exhibited increased vessel tone compared with *Jmjd3^fl/fl^Myh11^Cre–^* littermate controls in response to ET-1 (10^–7^ M), and this was negated by treatment with the dual endothelin receptor antagonist bosentan (10^–8^ M) (Figure 4A). This was also observed in mesenteric arteries, and although *Jmjd3^fl/fl^Myh11^Cre+^* mesenteric arteries displayed higher baseline contractility than *Jmjd3^fl/fl^Myh11^Cre–^* littermate controls, there was no difference in response to Ang II or PE (Figure 4B). This indicated that the effects of JMJD3 deletion on vessel tone were specific to endothelin signaling rather than other vasoactive agonists. In agreement with this, *Jmjd3^fl/fl^Myh11^Cre+^* aortas exhibited increased responsiveness to ET-1 stimulation compared with aortas isolated from *Jmjd3^fl/fl^Myh11^Cre–^* controls, and this enhancement in contractility was reduced to *Cre^–^* baseline levels following bosentan treatment (Figure 4C).

Next, to directly test the effect of JMJD3 deletion on SMC contractility, we utilized a collagen gel contraction assay in which aortic SMCs were isolated from *Jmjd3^fl/fl^Tagln^Cre^* mice and embedded in a collagen gel, treated with ET-1 (1 μM) and/or bosentan (10 μM), and gel area was measured. Gels containing *Jmjd3*-deficient mAoSMCs had smaller areas compared with control SMCs in response to ET-1, and treatment of gels containing SMCs deficient in *Jmjd3* with bosentan resulted in gel areas comparable to untreated gels (Figure 4D). Taken together, these results suggest that JMJD3 deletion results in increased SMC contraction via endothelin signaling, leading to increased arterial tone and BP.

Endothelin receptor stimulation results in ERK pathway activation, leading to SMC contraction (3, 39, 40). Supportive of this, treatment of HuAoSMCs with ET-1 (1 μM) for 5 minutes led to increased phosphorylated ERK (pERK), which was increased further by pretreatment of SMCs with the JMJD3 inhibitor GSKJ4 (50 nM) (Figure 4E). Next, we treated *Jmjd3^fl/fl^Tagln^Cre^* SMCs with ET-1 (1 μM) with or without the endothelin receptor antagonist bosentan (10 μM) and then measured pERK by Western blotting. As shown in Figure 4F, baseline ERK activity was modestly increased in Jmjd3-deficient SMCs compared with *Jmjd3^fl/fl^Tagln^Cre–^* SMCs, and treatment with ET-1 resulted in a substantial increase in pERK in Jmjd3-deficient SMCs compared with controls. Pretreatment of mAoSMCs with bosentan resulted in a small reduction in pERK activity in Jmjd3-deficient mAoSMCs. We also observed increased pERK in *Jmjd3^fl/fl^Tagln^Cre+^* SMCs compared with control SMCs via immunofluorescence (Figure 4G). Additionally, in immunofluorescence experiments, overexpression of flag-JMJD3 in mAoSMCs led to decreased pERK activity in mAoSMCs treated with Ang II (100 nM) (Figure 4H). Taken together, these results demonstrate that JMJD3 regulates endothelin-pERK signaling in SMCs.

**Figure 5:**
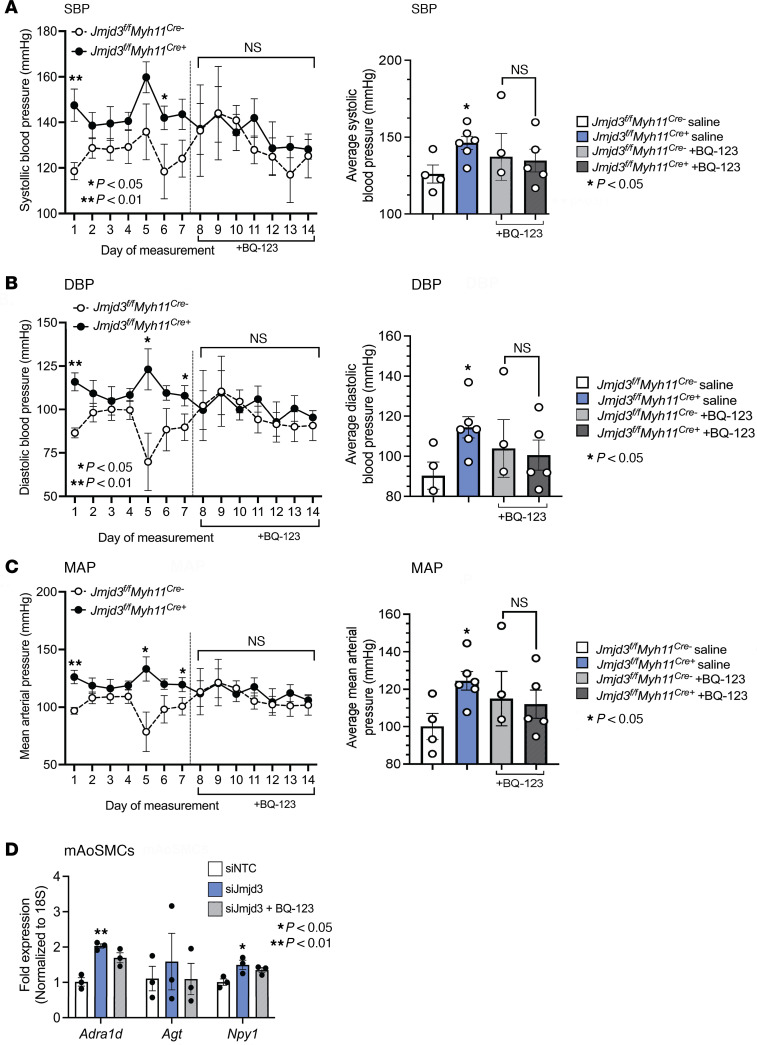
EDNRA antagonism normalizes BP after JMJD3 deletion in SMCs. BP of *Jmjd3^fl/fl^Myh11^Cre+^* and *Jmjd3^fl/fl^Myh11^Cre–^* mice treated with EDNRA inhibitor BQ-123 (200 nmol/kg/day) beginning on day 8 of measurements. SBP (**A**), DBP (**B**), and MAP (**C**). Values were also averaged the first 7 days and the final 7 days of the experiment (right of each figure). (**D**) qPCR for *Adra1d*, *Agt*, and *Npy1* in mAoSMCs transfected with siRNA to Jmjd3 or NTC and then treated with vehicle or BQ-123 (5 μM) for 16 hours. Data are represented as means ± SEM. *n* = 3 independent experiments. In vitro experiments representative of SMCs from 4–6 mice per group. Two-tailed Student’s *t* test. *P < 0.05; **P < 0.01.

To delineate the translational impact of enhanced endothelin signaling (specifically EDNRA) on BP after JMJD3 deletion in SMCs, we tested BP response in *Jmjd3^fl/fl^Myh11^Cre^* mice treated with the FDA-approved EDNRA-specific antagonist BQ-123. As observed above, *Jmjd3^fl/fl^Myh11^Cre+^* mice exhibited increased SBP, DBP, and MAP compared with *Cre^–^* littermate controls. However, treatment of *Jmjd3^fl/fl^Myh11^Cre+^* mice with BQ-123 (200 nmol/kg/day) normalized BP to *Cre^–^* levels (Figure 5, A–C). As above, knockdown of Jmjd3 in mAoSMCs led to upregulation of key HTN genes (see Supplemental Figure 4J), which was inhibited by treatment of mAoSMCs in vitro with BQ-123 (5 μM) (Figure 5D).

### JMJD3 is required for vascular SMC differentiation.

**Figure 6:**
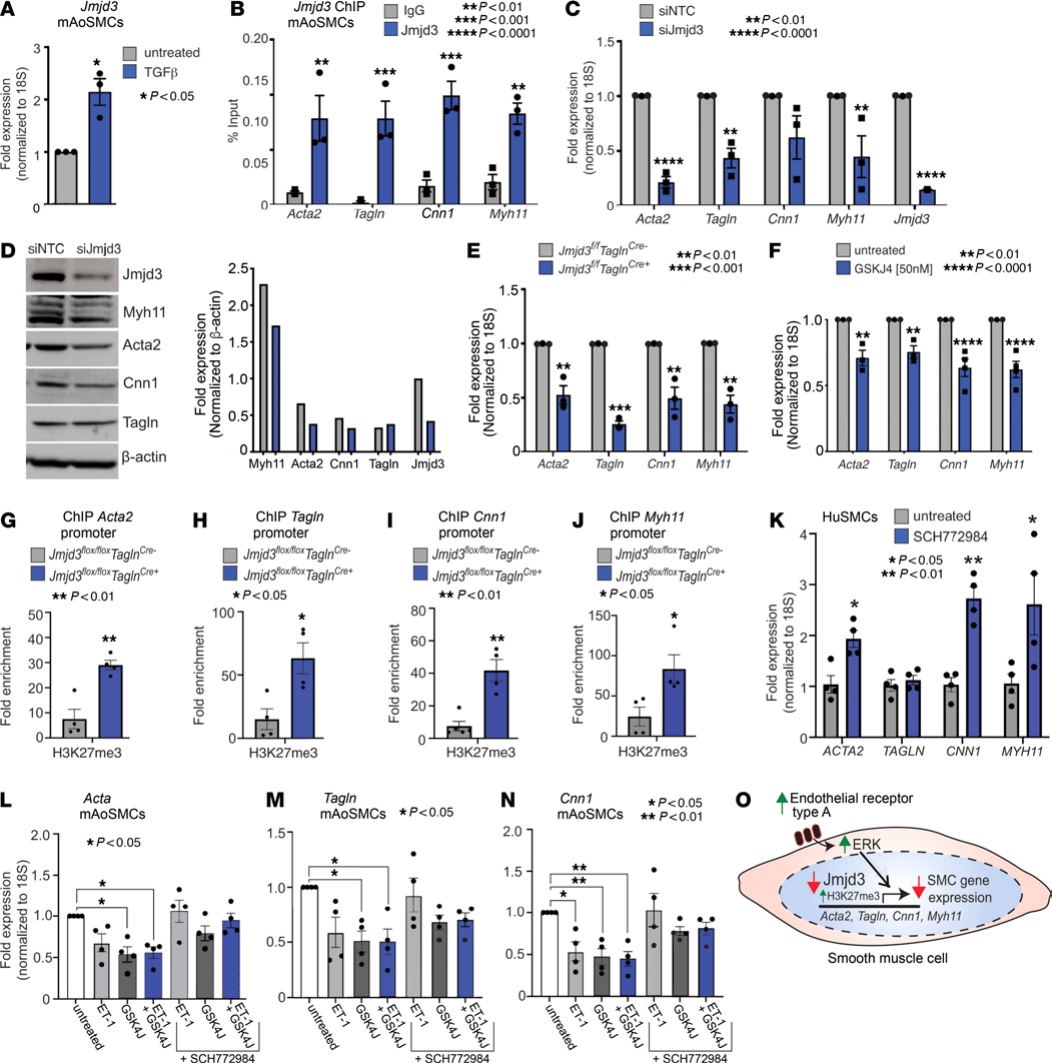
JMJD3 is required for vascular SMC differentiation. (**A**) qPCR of *Jmjd3* in mAoSMCs untreated versus treated with TGF-β (20 ng/ml) for 16 hours. (**B**) ChIP-qPCR showing Jmjd3 enrichment at SMC gene promoters compared with IgG. (**C**) SMC gene expression in mAoSMCs treated with Jmjd3 siRNA or NTC siRNA for 72 hours, serum starved, and then treated with TGF-β (20 ng/ml). (**D**) Representative Western blot probed for SMC markers in mAoSMC lysates treated with Jmjd3 or NTC siRNA. Representative densitometry of blot depicted to right. (**E**) qPCR of smooth muscle genes in *Jmjd3^fl/fl^Tagln^Cre^* mAoSMCs. (**F**) qPCR of *Acta2*, *Tagln*, *Cnn1*, and *Myh11* in mAoSMCs treated with Jmjd3 inhibitor GSKJ4 (50 nM) for 16 hours. (**G**) H3K27me3 ChIP-qPCR at *Acta2* (**G**), *Tagln* (**H**), *Cnn1* (**I**), *Myh11* (**J**) promoters in *Jmjd3^fl/fl^Tagln^Cre^* mAoSMCs. (**K**) qPCR for SMC genes in HuSMCs treated with SCH772984 (5 μM) for 16 hours compared with untreated. (**L**) qPCR for *Acta2* (**L**), *Tagln* (**M**), and *Cnn1* (**N**) in mAoSMCs treated with ET-1 (1 μM), GSK4J (50 nM), ET-1 with GSK4J with or without the ERK inhibitor SCH772984 (5 μM). (**O**) Illustration depicting effect of JMJD3 loss on SMC gene expression via EDNRA/ERK activation and H3K27me3 at SMC gene promoters. Data are represented as means ± SEM. *n* = 3 independent experiments. Tissues harvested from 4–6 mice per group. In vitro experiments representative of SMCs from 4–6 mice per group. One-way ANOVA (**L**–**N**) and 2-tailed Student’s *t* test (**A**–**C**, **E**, **F**, and **G**–**K**). *P < 0.05; **P < 0.01; ***P < 0.001; ****P < 0.0001.

During longstanding HTN, SMCs transition from a contractile to synthetic phenotype (41, 42). Given the role of JMJD3 in SMCs on BP in vivo, we investigated whether the SMC phenotype is altered by JMJD3. We examined mAoSMCs for *Jmjd3* expression after stimulation with TGF-β (20 ng/ml), since TGF-β is a well-established driver of SMC differentiation (11). TGF-β increased *Jmjd3* expression in mAoSMCs by approximately 2-fold (Figure 6A). Next, we performed ChIP for Jmjd3 on mAoSMCs at canonical SMC-specific gene promoters (*Acta2*, *Tagln*, *Cnn1*, *Myh11*). Jmjd3 demonstrated significant enrichment at smooth muscle gene promoters in mAoSMCs (Figure 6B). Next, we used siRNA-mediated knockdown of Jmjd3 in mAoSMCs treated with TGF-β (20 ng/ml) to investigate the effect of Jmjd3 on SMC-specific gene expression (*Acta2*, *Tagln*, *Cnn1*, *Myh11*). Jmjd3 knockdown reduced TGF-β–dependent expression of SMC genes (*Acta2*, *Tagln*, *Myh11*) at the mRNA and protein levels compared with control NTC siRNA (Figure 6, C and D). To examine this genetically, we analyzed smooth muscle gene expression in *Jmjd3^fl/fl^Tagln^Cre^* SMCs. SMC gene expression was significantly reduced in *Jmjd3^fl/fl^Tagln^Cre+^* SMCs compared with *Cre^–^* SMCs (Figure 6E). Further, pharmacologic treatment of mAoSMCs with the Jmjd3 selective inhibitor GSKJ4 (50 nM) inhibited smooth muscle gene expression (*Acta2*, *Tagln*, *Cnn1*, *Myh11*) (Figure 6F). To examine the direct effects of Jmjd3 on SMC gene promoters, ChIP was performed for H3K27me3 in mAoSMCs treated with siRNA to *Jmjd3* or NTC siRNA. Jmjd3 knockdown resulted in increased H3K27me3 enrichment at SMC-specific gene promoters (*Acta2*, *Tagln*, *Cnn1*, *Myh11*), indicating that JMJD3 positively regulates SMC gene expression directly by removing H3K27me3 from gene promoters (Figure 6, G–J). These results reveal that *JMJD3* expression is increased by TGF-β and required for SMC gene expression via an H3K27me3-mediated mechanism.

Given our findings that JMJD3 loss in SMCs increased endothelin signaling and ERK activation, as well as evidence from others demonstrating that increased ERK activity represses SMC gene expression, we explored whether JMJD3 controlled SMC gene expression via ERK signaling (43). First, as a translational corollary, treatment of HuSMCs with the ERK inhibitor SCH772984 (5 μM) robustly increased SMC gene expression (*ACTA2*, *CNN1*, *MYH11*) (Figure 6K). Next, to examine ERK inhibition after JMJD3 loss, we treated mAoSMCs with combinations of GSKJ4 (50 nM), ET-1 (1 μM), and/or SCH772984 (5 μM). ET-1 treatment markedly reduced SMC gene expression, which was further decreased by inhibiting Jmjd3 with GSKJ4 (50 nM). Treatment of mAoSMCs with SCH772984 (5 μM) prevented downregulation of SMC gene expression by ET-1 (1 μM) and GSKJ4 (50 nM) alone and in combination with each other (Figure 6, L–N). These data reveal that JMJD3 loss results in decreased smooth muscle gene expression via increased H3K27me3 at SMC gene promoters and by increased endothelin-ERK activation, inhibition of which restores SMC gene expression (Figure 6O).

### Hypertensive-induced arterial remodeling is regulated by JMJD3.

**Figure 7:**
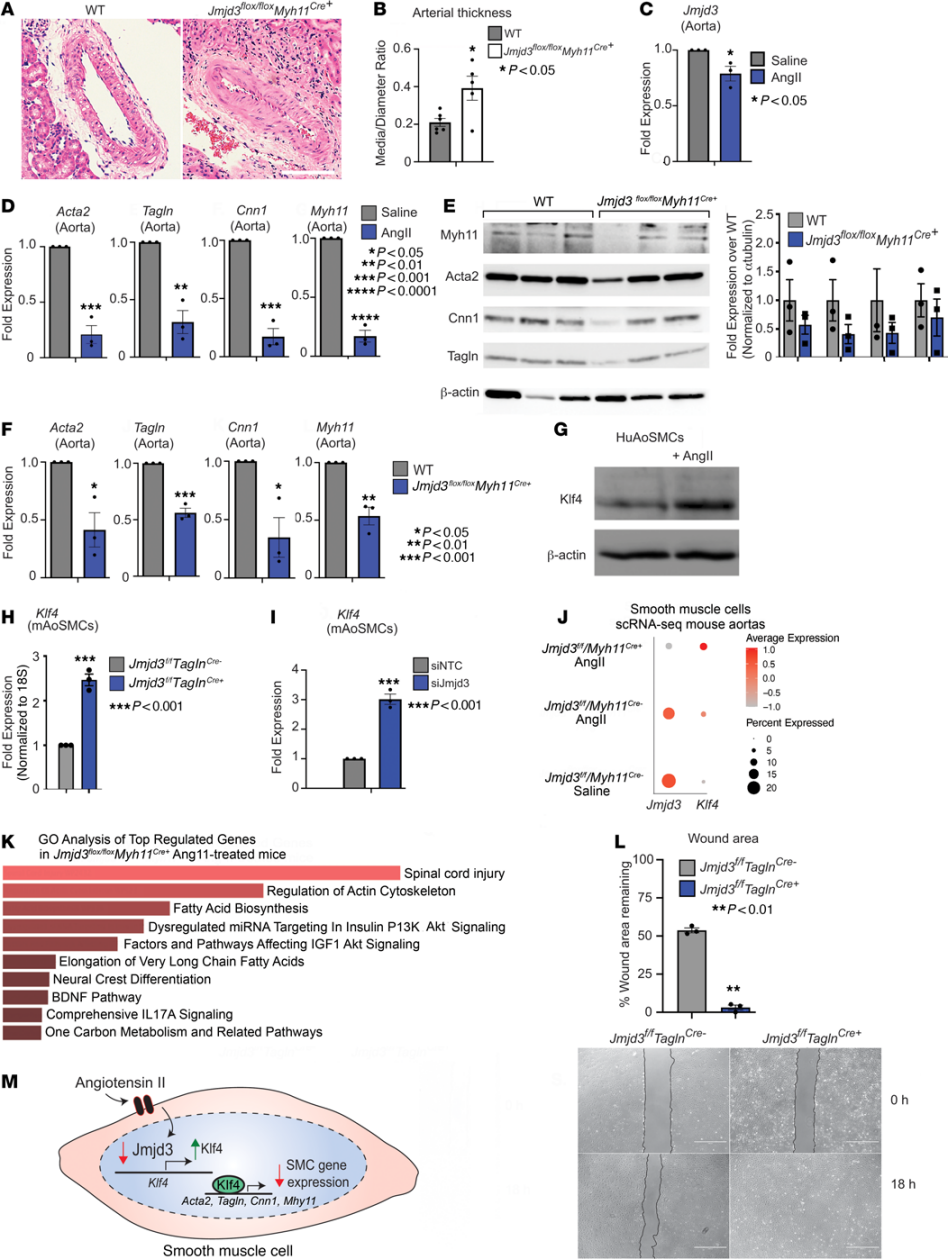
Hypertensive-induced arterial remodeling is regulated by JMJD3. (**A**) H&E staining of kidney sections showing renal arterioles from *Jmjd3^fl/fl^Myh11^Cre+^* and WT mice treated with Ang II (1 μg/kg/min) for 14 days. Scale bar: 100 μm. (**B**) Renal arteriole medial wall thickness from **A**. (**C**) qPCR for *Jmjd3* in WT aortas from mice treated with 14 days of Ang II or saline. (**D**) qPCR for *Acta2*, *Tagln*, *Cnn1*, and *Myh11* in aortas from mice in **C**. (**E**) Western blot for SMC markers from *Jmjd3^fl/fl^Myh11^Cre+^* and WT aortas with densitometry to right. (**F**) qPCR for *Acta2*, *Tagln*, *Cnn1*, and *Myh11* in aortas from *Jmjd3^fl/fl^Myh11^Cre+^* and WT mice treated with 14 days of Ang II. (**G**) Representative Western blot probed for KLF4 in HuAoSMCs serum starved or treated with Ang II for 16 hours. Blot representative of *n* = 3 independent experiments. (**H**) qPCR of *Klf4* expression in *Jmjd3^fl/fl^Tagln^Cre^* mAoSMCs. (**I**) qPCR of *Klf4* in mAoSMCs treated with NTC or siJmjd3 siRNAs and then serum starved for 16 hours. (**J**) Dot plot of *Jmjd3* and *Klf4* generated from scRNA-Seq of aortas isolated from *Jmjd3^fl/fl^Myh11^CreERT^* mice treated with saline or Ang II for 14 days (data representative of 2–3 mice per group). (**K**) Histogram of top-most upregulated gene pathways in aortic SMCs from **J**. (**L**) Results of scratch assay on *Jmjd3^fl/fl^Tagln^Cre^* mAoSMCs as percentage of wound area remaining after 18 hours following initial scratch. Representative images at 0 hours and 18 hours after scratch depicted on right. Scale bars: 1 mm. (**M**) Schematic of transcriptional regulation of Ang II/JMJD3/KLF4 axis controlling SMC gene expression. Data are represented as means ± SEM. *n* = 3 independent experiments. Tissues harvested from 4–6 mice per group. In vitro experiments representative of SMCs from 4–6 mice per group. Two-tailed Student’s *t* test. *P < 0.05; **P < 0.01; ***P < 0.001; ****P < 0.0001.

We show that JMJD3 regulates SMC differentiation and endothelin/ERK signaling, which both control SMC phenotype. Thus, we examined the role of JMJD3 in SMCs on arterial remodeling during longstanding HTN. Since BP is regulated by resistance arteries, we measured renal arteriole wall thickness from *Jmjd3^fl/fl^Myh11^Cre+^* and WT mice treated with Ang II for 14 days and observed increased media-to-diameter ratio in mice with SMC-specific deletion of Jmjd3 compared with littermate controls, indicating increased remodeling (i.e., increased migration and phenotypic modulation) (Figure 7, A and B). We assessed arterial beds from different vascular tissues under basal (saline) and Ang II–treated conditions and observed increased remodeling after Jmjd3 deletion in SMCs was most exaggerated in smaller resistance arteries (renal arterioles) in Ang II–treated mice and was overall unaffected in larger, conduit arteries (aorta) (Figure 7, A and B, and Supplemental Figure 6). Since Ang II induces SMC phenotypic modulation and arterial remodeling, we explored whether Ang II regulates JMJD3 expression, thereby leading to changes in SMC gene expression during HTN (41, 44). We first measured *Jmjd3* mRNA in aortas isolated from mice treated with saline or Ang II and observed decreased *Jmjd3* expression in aortas from Ang II–treated mice (Figure 7C). This was accompanied by reduced expression of *Acta2*, *Tagln*, *Cnn1*, and *Myh11* in whole aorta tissue (Figure 7D). Given that JMJD3 was required for SMC differentiation (see Figure 6), we determined whether JMJD3 regulated SMC gene expression during Ang II–mediated hypertensive arterial remodeling. We measured smooth muscle gene expression (*Acta2*, *Tagln*, *Cnn1*, *Myh11*) in *Jmjd3^fl/fl^Myh11^Cre+^* and WT mice treated with Ang II for 14 days. SMC-specific Jmjd3 deletion resulted in further loss of SMC markers (both mRNA and protein) in aortas from Ang II–treated mice (Figure 7, E and F). The TF KLF4 controls the SMC switch from the mature, contractile to the proliferative, synthetic phenotype; thus we determined whether *KLF4* expression was altered under hypertensive conditions (12, 15). Indeed, Ang II (100 nM) increased *KLF4* expression in HuAoSMCs in vitro, suggesting that KLF4 may drive phenotypic modulation during hypertensive remodeling (Figure 7G). Because loss of JMJD3 promoted the synthetic SMC phenotype, we tested whether KLF4 was transcriptionally regulated by JMJD3. First, we found that *Klf4* expression was upregulated 2.5-fold in *Jmjd3^fl/fl^Tagln^Cre+^* mAoSMCs compared with littermate *Cre^–^* controls (Figure 7H). Additionally, siRNA knockdown of Jmjd3 in mAoSMCs increased *Klf4* expression nearly 3-fold compared with NTC siRNA (Figure 7I). This was supported by scRNA-Seq of aortas from saline versus Ang II–treated *Jmjd3^fl/fl^Myh11^Cre^* mice, in which Ang II decreased *Jmjd3* expression and increased *Klf4* expression, which was further increased by Jmjd3 deletion (Figure 7J). Interestingly, GO analysis of differentially expressed genes (DEGs) in SMCs from these mice demonstrated that Jmjd3 loss in chronic HTN results in increased expression of genes related to tissue injury, actin cytoskeleton, AKT signaling, and inflammation, some of which are known downstream targets of ET-1 signaling (Figure 7K). SMC migration, which is regulated by ERK signaling and phenotypic modulation, was increased in *Jmjd3^fl/fl^Tagln^Cre+^* SMCs in scratch assays (smaller wound area remaining in *Cre^+^* SMCs) (Figure 7L). In summary, Ang II–induced downregulation of *JMJD3* in SMCs results in loss of mature SMC genes and increased *KLF4*, which cooperatively drive phenotypic modulation and remodeling during longstanding HTN (Figure 7M).

### JMJD3 regulates the contractile gene program in SMCs by cooperatively regulating SRF binding to SMC gene promoters.

**Figure 8:**
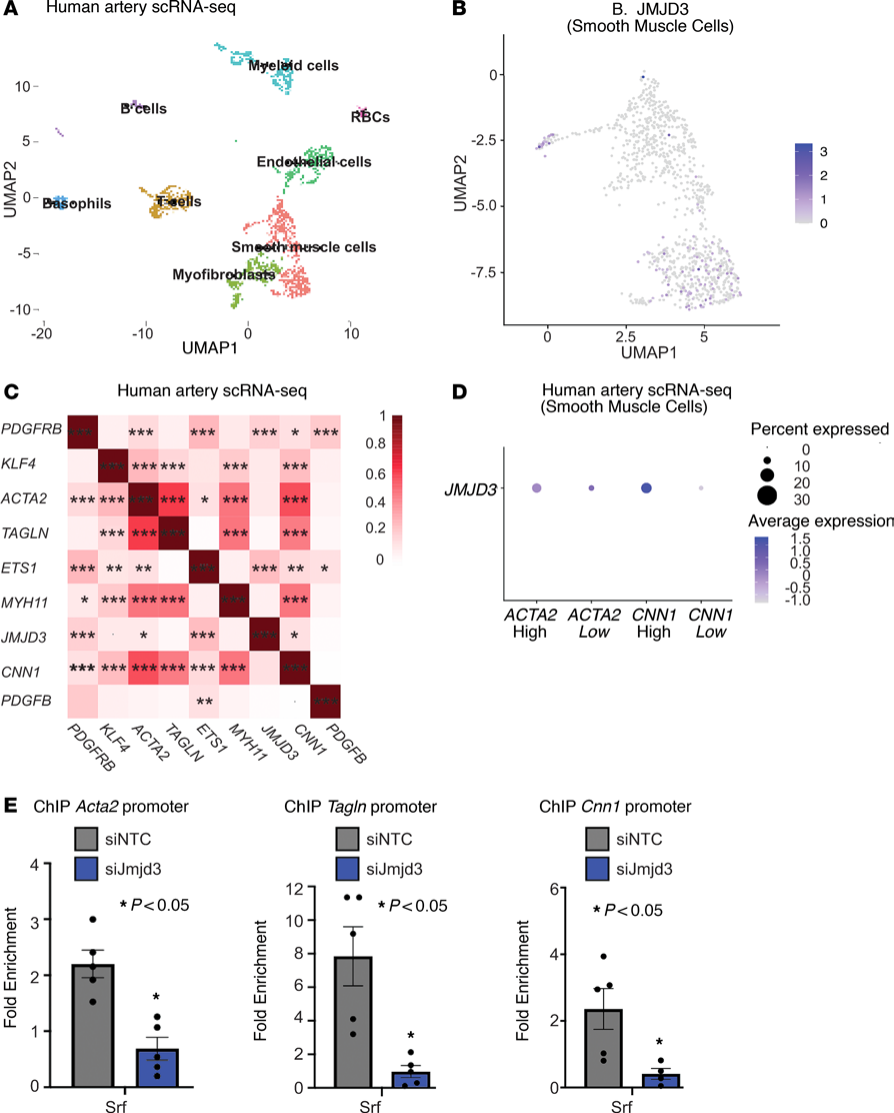
JMJD3 regulates the contractile gene program in SMCs by cooperatively regulating SRF binding to SMC gene promoters. (**A**) UMAP plots of cell populations from scRNA-Seq of human femoral arteries (*n* = 4 samples). (**B**) Relative expression of *JMJD3* in SMCs. (**C**) Graph of Pearson’s correlation of smooth muscle genes from scRNA-Seq of human arteries with heat gradient representing strength of association. (**D**) Dot plot of *JMJD3* in human artery SMCs separated by high versus low expression of *ACTA2* and *CNN1*. (**E**) Srf ChIP-qPCR using primers for *Acta2*, *Tagln*, and *Cnn1* promoters in mAoSMCs treated with NTC or siJmjd3 siRNA. Data are represented as means ± SEM. *n* = 3 independent experiments. *n* = 4 human arterial samples. Experiments representative of SMCs from 4–6 mice per group. Two-tailed Student’s *t* test and Pearson’s correlation coefficient. *P < 0.05; **P < 0.01; ***P < 0.001.

In order to translate the above in vivo murine findings to humans, we performed scRNA-Seq in human femoral artery samples (*n* = 4) (Figure 8A). First, we measured *JMJD3* expression in SMCs from human arteries and found *JMJD3* was expressed in SMCs at moderate levels (Figure 8B). Next, we performed Pearson’s expression correlation analysis among genes associated with contractile and synthetic gene programs in SMCs. As shown in Figure 8C, genes associated with mature, contractile SMC phenotype including *ACTA2*, *TAGLN*, *CNN1*, and *MYH11* showed very strong correlation with one another, yet weak or no correlation with synthetic, proliferative-associated genes including *PDGFBR*, *PDGFB*, *KLF4*, and *ETS-1*. Similarly, the proliferative genes exhibited strong correlation with each other. The distinct clustering of mature genes and proliferative genes confirmed the utility of this approach. Next, we performed analysis of *JMJD3* expression in SMC subsets separated into high or low expression of contractile genes. We found that *JMJD3* expression (as was the number of JMJD3-expressing cells) was increased in *ACTA2* and *CNN1* “high” SMCs compared with “low”-expressing SMCs (Figure 8D). Because CMEs cooperatively regulate TF binding, we investigated whether JMJD3 affected SRF binding to SMC gene promoters. We performed ChIP for Srf at SMC gene promoters in mAoSMCs treated with *Jmjd3* siRNA or NTC siRNA. We observed decreased binding of Srf to *Acta2*, *Tagln*, and *Cnn1* promoters in Jmjd3 knockdown mAoSMCs compared with control (Figure 8E). Taken together, our data reveal that JMJD3 regulates the contractile gene program in HuSMCs and cooperatively affects SRF binding at smooth muscle gene promoters.

## Discussion

Here, we define the mechanistic pathway linking the BP-associated polymorphism rs62059712 to transcriptional regulation of *JMJD3*, which regulates endothelin-dependent BP and SMC phenotype via epigenetic alterations at gene promoters and changes in ERK signaling. This work uncovers an allele-specific mechanism regulating expression of an epigenetic enzyme (JMJD3) that controls endothelin receptor expression and consequently, modulates SMC contractility, differentiation, and arterial remodeling in response to longstanding HTN. These findings identify a key pathway linking genetic and epigenetic mechanisms to molecular control of SMC function and phenotype, thereby revealing how GWAS and similar studies can be used to identify new epigenetic regulators of disease. The rs62059712 major T allele is associated with increased use of medications acting on the renin-angiotensin system, diuretics, and calcium channel blockers (Supplemental Table 1). Thus, the rs62059712 genotype-JMJD3 interaction may yield insight into genetic response to current antihypertensive therapies. Interestingly, the major T allele, which is associated with increased BP, is conserved across rodent species, indicating that the minor C allele confers a protective advantage despite its lower frequency in the population.

We characterize the effect of rs62059712 on *JMJD3* transcription, demonstrating that the minor C allele increases *JMJD3* promoter activity by creating an SP1-binding site within this region. SP1 loss phenocopied loss of JMJD3 in SMCs, indicating the important role of this TF in mediating downstream effects on gene expression. SP1 induces the SMC phenotypic switch after vascular injury by directly binding to GC repressor elements in SMC-specific genes (e.g., *MYH11*) as well as indirectly by increasing expression of *KLF4*, which inhibits myocardin function and downstream SMC differentiation (10, 12). In contrast, reports have demonstrated positive effects of SP1 on gene transcription, for example, increasing *ACTA2* expression in myofibroblasts (10). Furthermore, interactions involving other epigenetic complexes, such as p300 and acetylated histone 3, cooperatively regulate SP1 function to refine its transcriptional effect on SMCs (45). Thus, SP1 may control HTN gene expression via additional cooperative mechanisms, perhaps by interacting with SMAD2, which was partially investigated here. Additionally, JMJD3 influences SRF binding to SMC gene promoters, and identification of additional coregulators of JMJD3 will provide further insight into epigenetic regulation of the SMC phenotype in disease.

While SMCs are the main determinant of BP, other cell types play a role. In our study, despite JMJD3’s expression in other vascular cell types (e.g., myeloid cells), the allele-specific mechanism was only observed in SMCs, and loss of JMJD3 in non-SMC cell types did not affect BP. We used noninvasive measurements of BP, which we acknowledge can be variable. However, the differences in BP in Jmjd3-deficient versus WT mice were observed at individual days as well as when averaged over the course of the experiment. Further, repeat experiments corroborated our findings, demonstrating increased BP in mice with SMC-specific Jmjd3 loss.

BP regulation is controlled by numerous upstream molecular, genetic, and epigenetic signals. GWAS have provided insight into the genetic regulation of BP through identification of candidate variants associated with BP (46, 47). At least 3 SNPs have been identified that influence endothelin signaling. rs9349379, located in an intron within the *PHACTR1* gene, affects *ET1* expression by altering long-range gene interactions between *PHACTR1* and *ET1* (48). Additionally, rs1630736 in the *ET1* gene and rs10305838 in the *EDNRA* gene are associated with BP (49, 50). Here, we find that JMJD3 loss in SMC leads to increased endothelin-ERK signaling, resulting in decreased SMC gene expression. Our results suggest that targeting ERK in longstanding HTN, and perhaps other models of vascular injury, may limit pathologic arterial remodeling. Keaton et al. reported a 0.47 mmHg increase in SBP for each copy of the major allele (29). While this per-copy allele effect is seemingly small, albeit typical for similar reported SNPs, it is likely that this SNP interacts with other genetic, epigenetic, and molecular signals to create larger changes in BP.

While prior studies have investigated the role of epigenetic alterations in cardiovascular disease, the exact role of H3K27me3 in HTN remains unknown (20–25, 27). Increased H3K27me3 levels have been associated with BP (51). We show that H3K27me3 enrichment at *EDNRB*, *ACTA2*, *TAGLN*, *CNN1*, and *MYH11* gene promoters is regulated by JMJD3, and future studies will determine how H3K27me3 at these promoters changes in the setting of HTN. Our identification of *EDNRB* as a direct transcriptional target of JMJD3 is consistent with reports that have identified that *EDNRB* expression is positively regulated by SMC-specific TFs (e.g., MKL2) (52). While SMCs are the predominant cell type regulating vessel tone and BP, endothelin signaling involves interplay between SMCs and ECs. Therefore, JMJD3 in SMCs and ECs may synergistically regulate arterial remodeling during HTN. Finally, deletion of JMJD3 led to increased HTN-associated genes, including ET-1 (*EDN1*). Thus, loss of JMJD3 may create a feed-forward mechanism whereby increased ET-1 and other HTN-associated genes worsen HTN.

We demonstrate that the rs62059712 major T allele decreases *JMJD3* expression, mechanistically resulting in a “double hit,” first leading to increased BP via increased *EDNRA* expression, and next, resulting in increased pathologic remodeling due to increased ERK signaling and H3K27me3 at smooth muscle gene promoters. In our analysis of different vascular beds, we observed notable differences in media thickness in Ang II–treated mice, which was not as prominent under basal conditions. This suggests that regulation of arterial remodeling by JMJD3 requires a “second hit,” such as prolonged HTN. Although we observed differences in downstream gene expression and BP under basal conditions (i.e., saline treatment), this did not translate to changes in remodeling the phenotype under basal conditions.

BP is a complex phenotype controlled by multiple factors. Our study reveals that the rs62059712 major T allele decreases *JMJD3* transcription in SMCs by disrupting SP1 binding to the *JMJD3* promoter, leading to decreased *JMJD3* expression. We identify the *EDNRB* gene as a direct transcriptional target of JMJD3, which is decreased by the major T allele, thereby resulting in compensatory increase in *EDNRA* expression. Thus, loss of *JMJD3* increases endothelin signaling and downstream vessel contractility. Specific inhibition of EDNRA receptor normalized the increased BP resulting after JMJD3 loss in SMCs. Decreased *JMJD3* further negatively modifies disease phenotype by leading to increased ERK activation, which increases SMC migration and phenotypic modulation during pathologic arterial remodeling. In conclusion, our findings define a unique transcriptional and molecular axis involving the histone demethylase JMJD3 in SMCs that regulates BP and arterial response to injury.

## Methods

### Sex as a biological variable.

For BP experiments using *Jmjd3^fl/fl^Myh11^CreERT^* mice, only male mice were used because the *Myh11^CreERT^* transgene is located on the Y chromosome.

### Animals.

Mice were housed in stock-holding rooms under pathogen-free conditions. *Jmjd3^fl/fl^Myh11^CreERT^*, *Jmjd3^fl/fl^Tagln^Cre^*, *Jmjd3^fl/fl^Cdh5^CreERT^*, and *Jmjd3^fl/fl^Lyz2^Cre^* mice were bred on a C57BL6/J background, and experiments were performed on 8- to 12-week-old mice. For inducible deletion of *Jmjd3* in SMCs and ECs, mice were injected intraperitoneally with tamoxifen (75 mg/kg) for 5 consecutive days, then allowed a 3-day washout period before experiments. Mice had free access to water and food throughout the study.

### Ang II infusion and noninvasive BP measurements.

Osmotic mini-pumps (ALZET, Model 2004) containing saline or Ang II were inserted subcutaneously in mice as previously described (30). Ang II was infused at a rate of 1 μg/kg/min. Mice were allowed to recover for 1 day before measuring BP. Daily BP measurements were obtained using a noninvasive tail-cuff system (Kent Scientific). Animals were allowed to equilibrate in the system, and at least 2 cycles of preliminary BP measurements were performed to ensure accurate recording. BP was measured daily through day 14, at which time mice were sacrificed and tissues harvested for downstream analysis. In EDNRA inhibitor experiments, mice were treated with BQ-123 (200 nmol/kg/day) each day beginning on day 8 of BP measurements.

### Aortic and mesenteric artery ring contractility assays.

Aortas and mesenteric arteries were isolated after 5 days of intraperitoneal tamoxifen (75 mg/kg) or corn oil injection of *Jmjd3^fl/fl^Myh11^CreERT^*mice, following a 3-day washout period. For each mouse, vessels were cut into 2 rings and run in parallel. Aortic and mesenteric rings were mounted vertically on 2 wire hooks and immersed in 2 ml KH buffer (118 mM NaCl, 4.8 mM KCl, 1.2 mM MgSO4, 1.2 mM KH2PO4, 25 mM NaHCO3, 11 mM glucose, and 2.5 mM CaCl2; pH 7.4) maintained with 95% O2–5% CO_2_ at 37°C. Vessel rings were equilibrated for 90 minutes with a resting tension of 1 g. After baseline tension was determined, vessels were precontracted with 60 mM KCl. Increasing concentrations of ET-1 (10^–10^–10^–7^ M), Ang II (10^–9^–10^–5^ M), and PE (10^–9^–10^–4^ M) were used. Concentration of bosentan used was 10^–8^ M. Values were normalized to percentage contraction of KCl.

### Cell culture.

Human aortic and bronchial SMCs were purchased from Lonza and maintained in Clonetics Smooth Muscle Growth Medium-2 (SMGM-2) supplemented with growth factors, 5% FBS, and antibiotics. HuSMCs were maintained and used for experiments between passages 2 and 14. Primary mAoSMCs were isolated from thoracic aortas of 12- to 16-week-old mice and maintained through passage 14 in DMEM F12 with 10% FBS and 0.5% penicillin and streptomycin.

### Plasmids.

pGL3-*JMJD3* DHS 1 and 2 major and minor allele reporter constructs were generated by amplifying 600 bp gene regions from a HuSMC genomic DNA template. DNA fragments were then cloned into pGL3 luciferase basic vector (Addgene), and the correct sequence was verified by Sanger sequencing. Mutations were introduced using the QuikChange Site-Directed Mutagenesis protocol (Agilent). All mutations were verified by Sanger sequencing. pCS2-Jmjd3-F was a gift from Kai Ge (Addgene plasmid 17440; http://n2t.net/addgene:17440).

### Luciferase assays.

mAoSMCs were seeded in 24-well plates at 2.4 × 10^4^ cells/well. Cells were transfected the day after plating with 50 ng of plasmid per well. Luciferase activity was measured 48 hours after transfection using the Steady-Glo Luciferase Kit (Promega) according to the manufacturer’s instructions. Raw luciferase values were normalized to the activity of the pGL3 empty vector.

### Gel contractility assays.

mAoSMCs were trypsinized when 80% confluent and resuspended at 6 × 10^5^ cells/mL in DMEM F12 with 10% FBS and antibiotics. Cells were diluted in type 1 collagen with DMEM F12 with 10% FBS and 0.5% antibiotics to 1 mg/mL collagen and 3 × 10^5^ cells/mL final concentration; 500 μL of the mixture was added to each well of a 24-well plate. Gels were allowed to polymerize at 37°C for 1 hour. Once gels were polymerized, gels were freed from well edges and 500 μL of medium (with or without pharmacologic agent) was added to each well. Collagen gels were incubated for 24 hours, and the area of each gel was measured over time.

### Scratch assays.

Cells were seeded in a 6-well culture dish and grown to 90% confluence. Cells were treated with 5 μg/ml mitomycin C for 2 hours. A vertical scratch was made in each well with a p200 pipette tip, the media was removed, washed once with PBS, and fresh media was added. Scratch area was measured from 0 hours to 18 hours.

### siRNA knockdowns.

mAoSMCs were transfected with 40 nM siRNA (Dharmacon) targeted to *Jmjd3*, *Sp1*, *Ednrb*, or a NTC siRNA for 72 hours. RNAiMax (Invitrogen) was used as the transfection reagent. Depending on the experiment, cells were either left in 10% FBS media, serum starved, or serum starved then treated with agonist or pharmacologic agent.

### RNA extraction and qRT-PCR.

Cultured SMCs were collected directly in TRIzol and RNA was extracted and converted to cDNA using the Superscript cDNA Synthesis Kit (Invitrogen); 20–50 ng cDNA was used in downstream quantitative real-time TaqMan PCR (qRT-PCR) and normalized using 18S. Tissues were snap frozen, pulverized using mortar and pestle, and collected in TRIzol (Invitrogen). Homogenized tissue was digested into single-cell suspension using a 20-gauge needle and syringe. Samples underwent RNA extraction and downstream qRT-PCR as above.

### Western blotting.

Cultured SMCs were lysed in RIPA buffer plus protease and phosphatase inhibitors and 1 mM dithiothreitol (DTT). Tissues were snap frozen, pulverized, and collected in RIPA buffer with protease and phosphatase inhibitors and 1 mM DTT as above. Lysates were normalized by total protein concentration, sample buffer was added, and then samples were boiled for 5 minutes; 10 to 50 μg of protein was loaded on an SDS-PAGE gel and run. Proteins were transferred to nitrocellulose or PVDF membranes and blocked in either 3% BSA or 5% nonfat dry milk. Blocked membranes were incubated with primary antibodies overnight at 4°C. Membranes were washed and incubated with species-specific HRP-linked secondary antibodies, washed, and developed. For densitometry results, Western blots were analyzed using NIH ImageJ software (NIH).

### Affinity purification of TFs.

Human aortic SMC nuclear lysates were prepared using the NUPER Nuclear Extraction Kit (Thermo Scientific) per the manufacturer’s protocol. Nuclear extracts were diluted with 2 volumes of 1.3× PD buffer (1×: 10mM Hepes pH 7.4, 8% glycerol, 1 mM MgCl2, 0.05% Triton X-100, 1.3 mM DTT, protease and phosphatase inhibitors). 5′ Biotinylated sense and unmodified anti-sense 25 bp oligos (Sigma) were annealed, added to diluted precleared nuclear lysates, and incubated with rotation for 10 minutes at room temperature. Streptavidin Dynabeads (Invitrogen) were added to the mixture, incubated for 30 minutes with rotation, and then beads were washed. Complexes were eluted in 2× sample buffer for analysis via SDS-PAGE and Western blotting.

### ChIP experiments.

mAoSMCs were fixed for 10 minutes in 0.7% formaldehyde. The crosslinking reaction was quenched by incubating cells with 0.125 M glycine for 5 minutes. ChIP assays were performed using the Abcam ChIP kit (Abcam catalog ab500) according to the manufacturer’s instructions. Sheared chromatin was incubated overnight at 4°C with 2 μg of JMJD3 antibody (Abcam), H3K27me3 antibody (Active Motif), or normal rabbit IgG antibody (Diagenode). Eluted DNA was used in downstream qPCR assays.

### Histology and immunofluorescence.

Tissues were harvested from mice and fixed in 10% formalin for 24 hours, then stored in 70% ethanol. Specimens were embedded in paraffin and sectioned onto microscope slides. After deparaffinization, sections underwent H&E staining or were processed for immunofluorescence. For immunofluorescent staining, slides underwent antigen retrieval in citric acid buffer (pH 6.0). Samples were then permeabilized, blocked, and incubated with primary antibody in blocking solution overnight at 4°C in a humidity chamber. Specific antibodies with dilutions and source are listed separately in Supplemental Table 2. The following day, slides were washed in PBS, then incubated with fluorophore-conjugated secondary antibody (1:500) in PBS for 2 hours at room temperature. Slides were washed with PBS, mounted, allowed to dry overnight, and then imaged.

### RNA-Seq experiments.

Cultured SMCs from *Jmjd3^fl/fl^Tagln^Cre^* mice were plated in 6-well plates at approximately 60% confluence. Cells were harvested the day after initial plating. Three biological replicates for each genotype were used. RNA isolation was performed using RNeasy Kit (QIAGEN) with DNAse digestion. Library construction and analysis of reads were performed as described previously (53). Briefly, reads were trimmed using Trimmomatic and mapped using HiSAT2 (54, 55). Read counts were performed using the feature-counts option from the subRead package followed by the elimination of low reads, normalization, and differential gene expression using edgeR (56, 57). Differential expression was performed on mapped reads using the Taqwise dispersion algorithm in edgeR.

### Phenome-wide association study.

OpenGWAS (https://gwas.mrcieu.ac.uk/), a database of 350 billion genetic associations from 50,044 GWAS summary datasets, and the GWAS Catalog (https://www.ebi.ac.uk/gwas/), a repository of GWAS summary statistics maintained by National Human Genome Research Institute and European Bioinformatics Institute, were queried for the lead candidate variant, rs62059712. Summary statistics were downloaded and compiled. Phenotypes associated with rs62059712 were noted.

### scRNA-Seq experiments.

Generation of single-cell suspensions for scRNA-Seq was performed as described by our group previously (31). Briefly, femoral artery specimens were harvested during femoral endarterectomy, femoral-femoral bypass, or aorto-bi-femoral bypass operations. Samples were digested overnight at 4°C. Cells were strained and then combined in a 1:1 ratio for scRNA-Seq by the University of Michigan Advanced Genomics Core on the 10x Genomics Chromium System. Libraries were sequenced on the Illumina NovaSeq 6000 sequencer. NovaSeq was used as the sequencing platform to generate 151 bp paired-end reads. We conducted adapter trimming and quality control procedures as described previously (58). The reads were then mapped using STAR (59) to build human GRCh37, and gene expression levels were quantified and normalized by HTSeq (60) and DESeq2 (61), respectively. Negative binomial models in DESeq2 were used to conduct differential expression analysis. Data processing, including quality control, read alignment, and gene quantification, was conducted using the 10x Genomics Cell Ranger software. Seurat was then used for normalization, data integration, and clustering analysis (62). All clustered cells were mapped to corresponding cell types by matching cell-cluster gene signatures with putative cell-type–specific markers.

### Statistical fine mapping.

GWAS summary statistics from Keaton et al. (29) were subsetted to include a 500 kb region on chromosome 17 centered about the GWAS lead variant rs62059712 (chr17:7490170-7990170). Statistical fine mapping using Bayes factor analysis was performed for a 99.9% credible set in R using the function calc_credset from the package levinmisc (https://github.com/mglev1n/levinmisc/blob/main/R/calc_credset.R).

### Statistics.

GraphPad Prism software (RRID:SCR_002798), version 9.2.0, was used to analyze the data. All data were analyzed for normal distribution and then statistical significance between multiple groups was obtained using two-tailed Student’s *t* tests, ANOVA, or Pearson’s correlation where appropriate. All *P* values less than or equal to 0.05 were considered significant.

### Study approval.

All experiments using human samples were approved by the IRB at the University of Michigan (IRB #: HUM00098915) and were conducted in accordance with the principles in the Declaration of Helsinki. Subjects gave informed consent. Animal studies were approved by the Animal Care Committee of the University of Michigan and complied with the NIH *Guide for the Care and Use of Laboratory Animals* (National Academies Press, 2011).

### Data availability.

Sequencing data are available in the NCBI’s Gene Expression Omnibus database (GEO GSE292027 and GSE292037). Values for all data points in graphs are reported in the Supporting Data Values file. Data are available from the corresponding author upon request.

## Author contributions

KDM and KAG conceived the project. KDM, LC, ATO, FMD, LCT, JG, KAG, and SMD designed experiments. KDM, QL, KH, TMB, SJW, JS, JYM, ECB, ADJ, ZA, RW, KB, and GSDJ performed experiments. KDM, QL, KH, ADJ, RW, LCT, and SMD analyzed data. KDM, QL, KH, and SMD created the figures. KDM, KAG, and SMD wrote the manuscript. KDM and KAG acquired funding.

## Supplementary Material

Supplemental data

Unedited blot and gel images

Supplemental table 1

Supplemental table 2

Supporting data values

## Figures and Tables

**Table 2:**
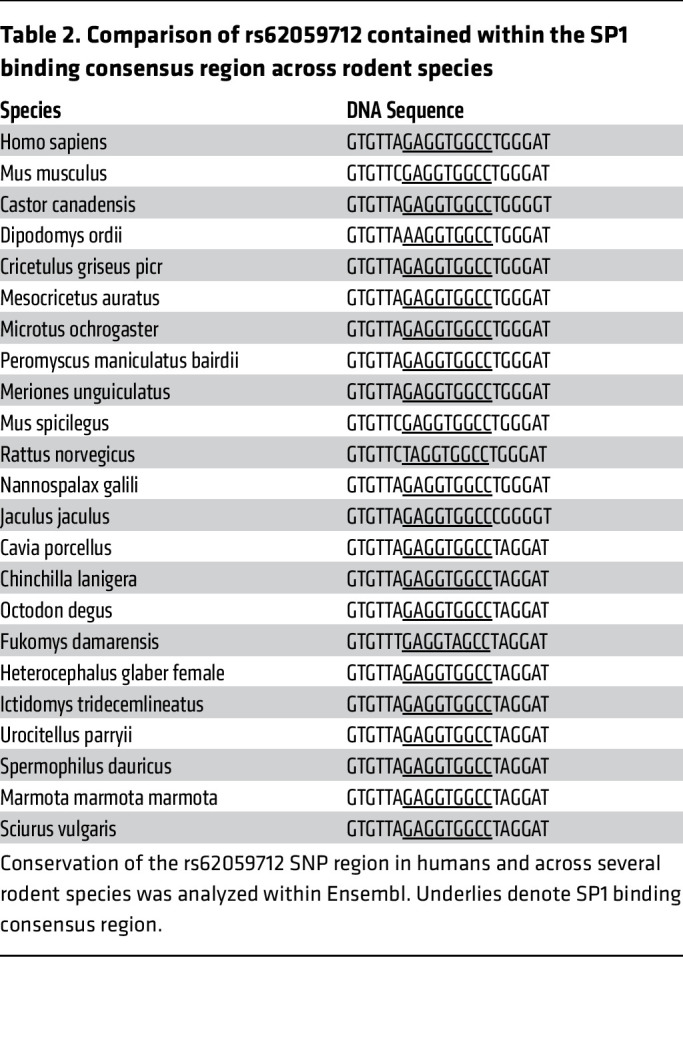
Comparison of rs62059712 contained within the SP1 binding consensus region across rodent species

**Table 1:**
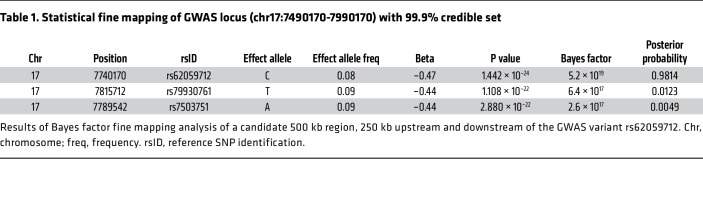
Statistical fine mapping of GWAS locus (chr17:7490170-7990170) with 99.9% credible set

## Version 1 03/27/2025

In-Press Preview

## Version 2 06/02/2025

Electronic publication
