# Supplementary material for: Epigenetic alteration of smooth muscle cells regulates endothelin-dependent blood pressure and hypertensive arterial remodeling
Source: J Clin Invest. 2025 Mar 27;135(11):e186146. doi: 10.1172/JCI186146 (PMC12126237; doi:10.1172/JCI186146)
Supplement: Supplemental data [file jci-135-186146-s107.pdf]

Supplemental Figures with Legends

Supplemental Figure 1

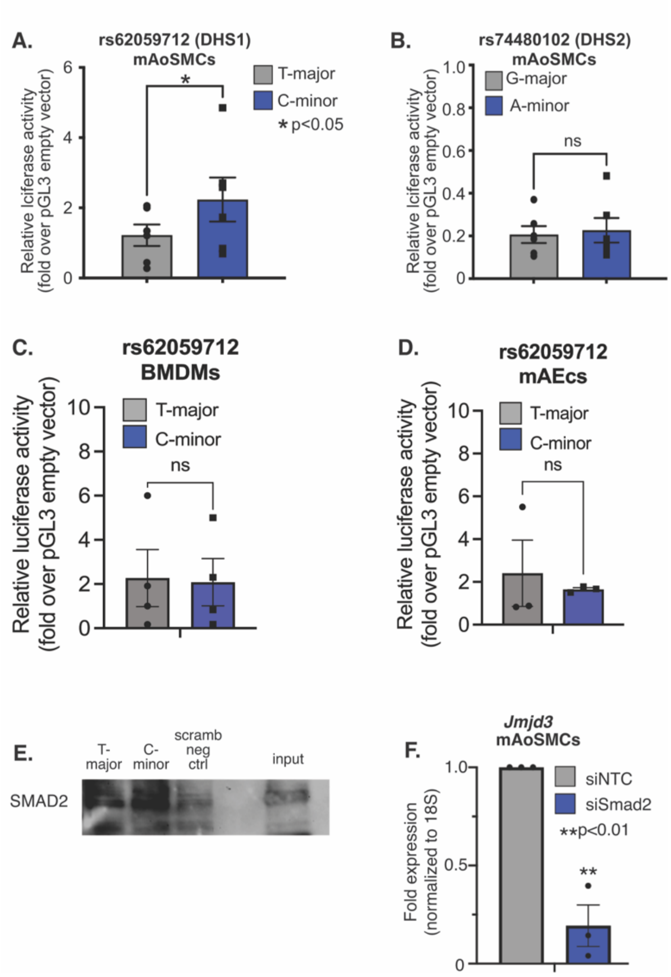

Supplemental Figure 1. A) Luciferase results of the DHS1 containing rs62059712 (T-major vs. C-minor) and B) DHS2 containing rs74480102 (G-major vs. A-minor) in cultured primary mAoSMCs. C) Luciferase results of rs62059712 T-major and C-major promoter constructs in BMDMs and (D) in mouse aortic endothelial cells (mAECs). E) Western blot of Smad2 after affinity purification using T vs. C probes corresponding to rs62059712 SNP region incubated with human aortic SMC nuclear lysate and then resolved by western blotting. F) qPCR for *Jmjd3* expression after NTC vs. Smad2 knockdown in mAoSMCs. Data are presented as the mean ± SEM, n=3 independent experiments. Experiments representative of SMCs from 4-6 mice per group. Two-tailed Student's t-test was used. *p<0.05, **p<0.01.

Supplemental Figure 2

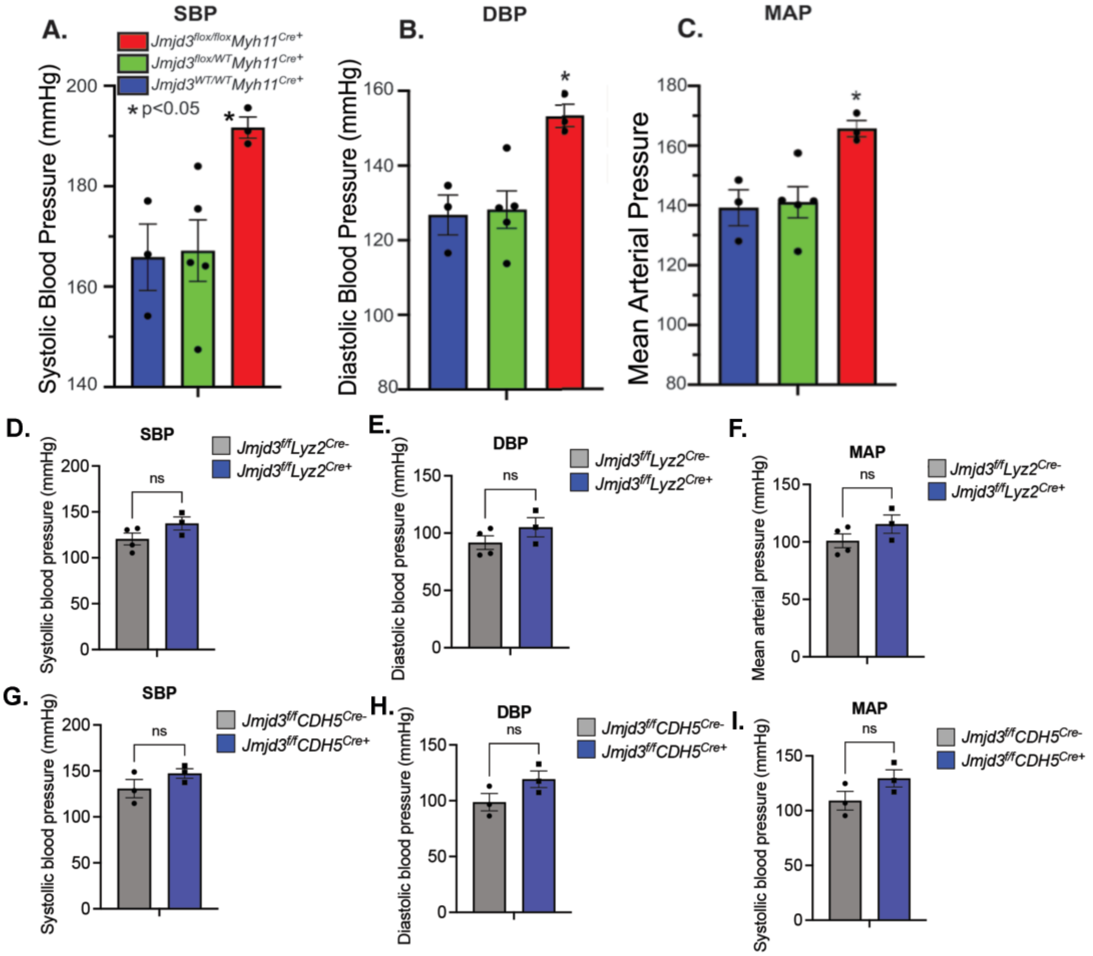

Supplemental Figure 2. A) Averages of systolic blood pressure (SBP), B) diastolic blood pressure (DBP), and C) mean arterial pressure (MAP) over 14 days in *Jmjd3*$^{flox/flox}$*Myh11*^{CreERT} mice homozygous, heterozygous, or absent (WT) for the floxed allele injected with tamoxifen (75 mg/kg) and infused with Angiotensin II (1ug/kg/min). D) SBP, E) DBP, and F) MAP obtained by tail cuff were averaged in *Jmjd3*$^{flox/flox}$Lyz2^{Cre} mice. G) SBP, H) DBP, and I) MAP were averaged in *Jmjd3*$^{flox/flox}$CDH5^{Cre} mice. Data are presented as the mean ± SEM. Results are averaged from 3-6 mice per group. Two-tailed Student's t-test was used to compare blood pressures from WT vs. *Jmjd3*$^{flox/flox}$*Myh11*^{CreERT} mice; *Jmjd3*$^{flox/flox}$Lyz2^{Cre}; and *Jmjd3*$^{flox/flox}$CDH5^{CreERT} mice, *p<0.05.

Supplemental Figure 3

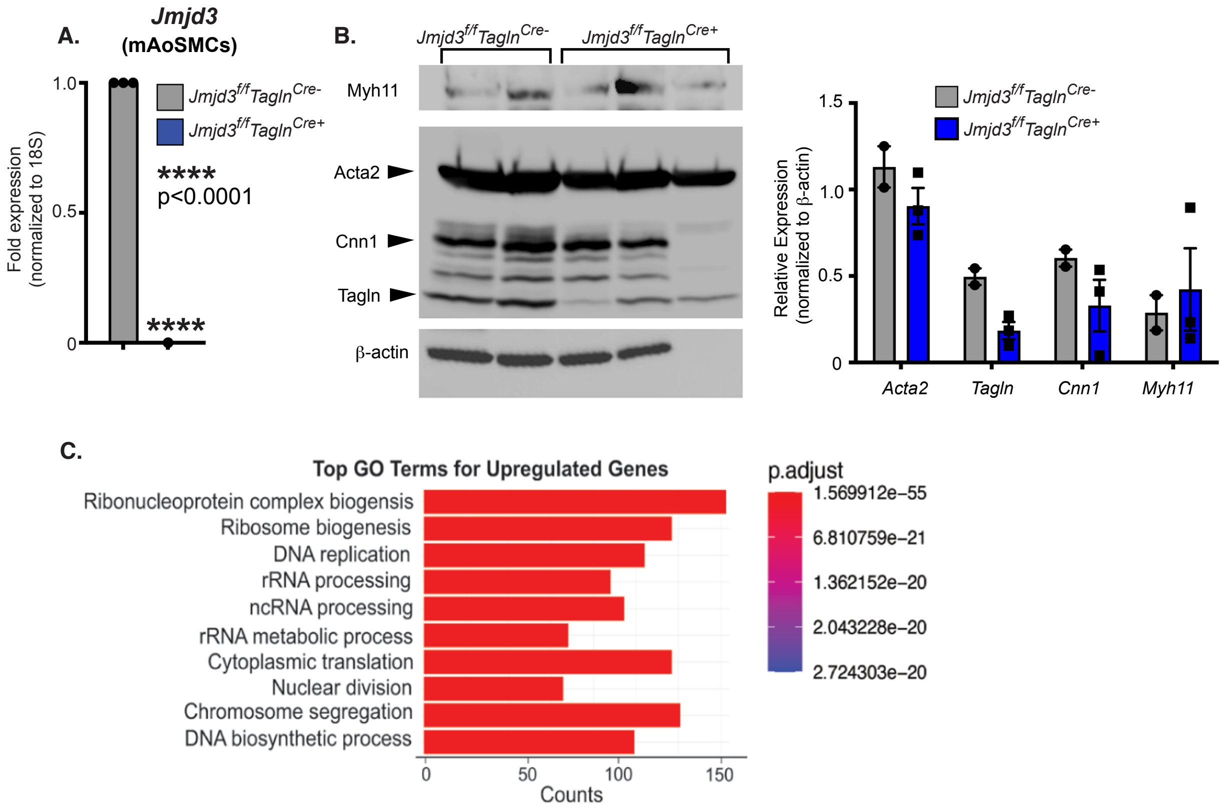

Supplemental Figure 3. A) qPCR of *Jmjd3* expression in SMCs isolated from *Jmjd3*$^{flox/flox}$*Tagln*^{Cre} mice. B) Western blot of aortas isolated from *Jmjd3*$^{flox/flox}$*Tagln*^{Cre} mice probed for SMC markers with accompanying densitometry results to right. C) Bar graph of gene ontology (GO) analysis for top 10 upregulated genes in *Jmjd3*$^{flox/flox}$ *Tagln*$^{Cre+}$ SMCs from RNA-seq results. Gene pathways listed on y-axis and number of gene counts for each pathway are listed on x-axis. Data are presented as the mean ± SEM, n=3 independent experiments, n=4-6 mice per group. Two-tailed Student's t-test, ****p<0.0001.

Supplemental Figure 4

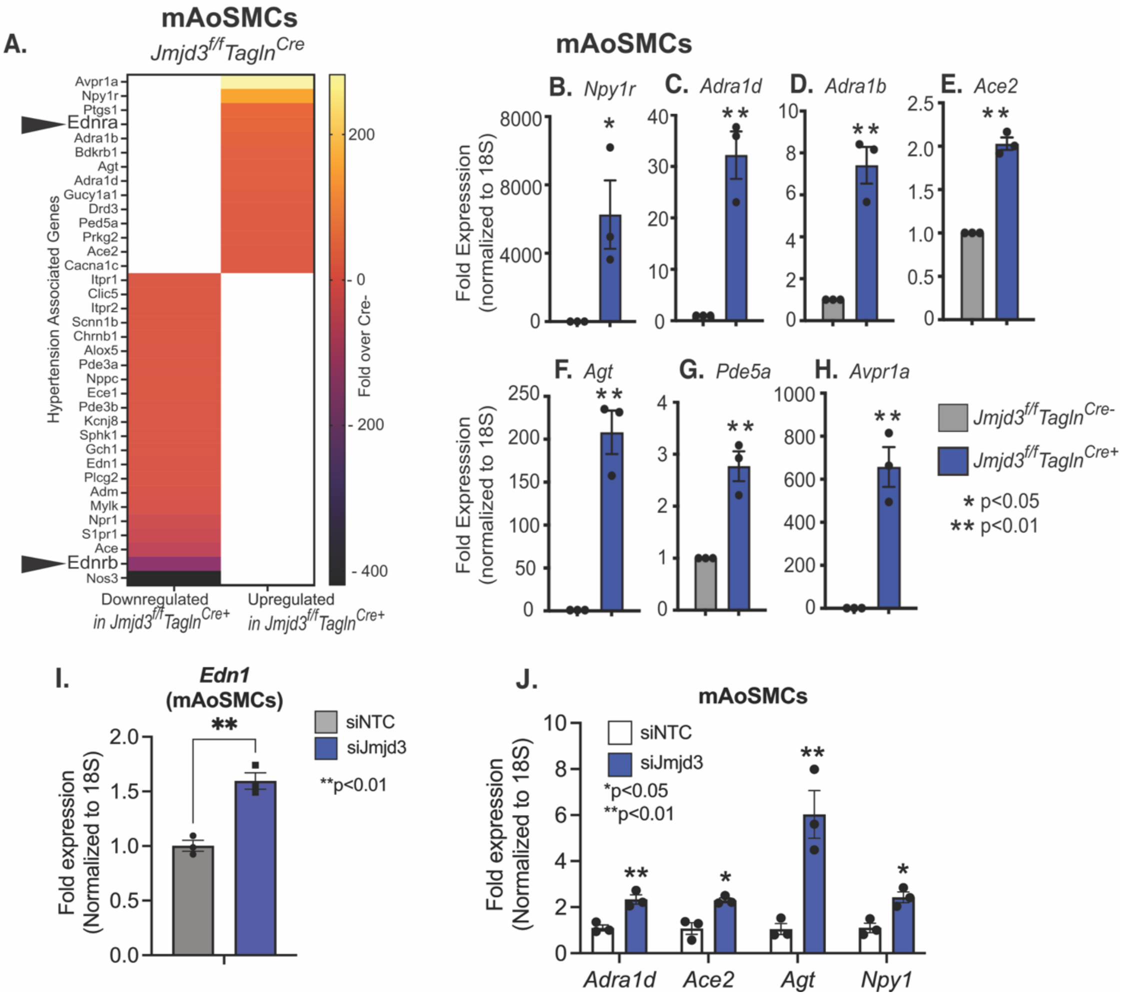

Supplemental Figure 4. A) Superarray heatmap comparing differentially expressed hypertension-associated genes in mAoSMCs from *Jmjd3*$^{flox/flox}$*Tagln*$^{Cre+}$ mice compared to *Jmjd3*$^{flox/flox}$*Tagln*$^{Cre-}$ mice. Data presented as fold over gene expression values in *Jmjd3*$^{flox/flox}$*Tagln*$^{Cre-}$ SMCs. *Ednra* and *Ednrb* designated with arrowheads. B) Validation qPCR for several of hypertension genes in A) including *Npy1r* (B), *Adra1d* (C), *Adra1b* (D), *Ace2* (E), *Agt* (F), *Pde5a* (G), and *Avpr1a* (H). I) qPCR for endothelin-1 (Edn1) in mAoSMCs treated with siRNA to Jmjd3 or siNTC and then serum starved for 16 hours. J) qPCR for Adra1, Ace2, Agt, and Npy1 in mAoSMCs treated with siRNA to Jmjd3 or siNTC. Data are presented as the mean ± SEM, n=3 independent experiments. Results represent data from SMCs from 4-6 mice per group. Two-tailed Student's t-test, *p<0.05, **p<0.01.

Supplemental Figure 5

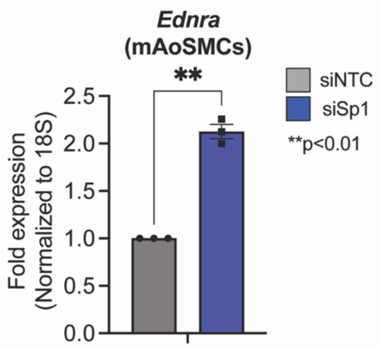

Supplemental Figure 5. qPCR for *Ednra* expression after NTC vs. Sp1 knockdown in mAoSMCs. Data are presented as the mean ± SEM, n=3 independent experiments. Data were first analyzed for normal distribution, and if data passed the normality test, a two-tailed Student's *t* test was used, **p<0.01.

Supplemental Figure 6

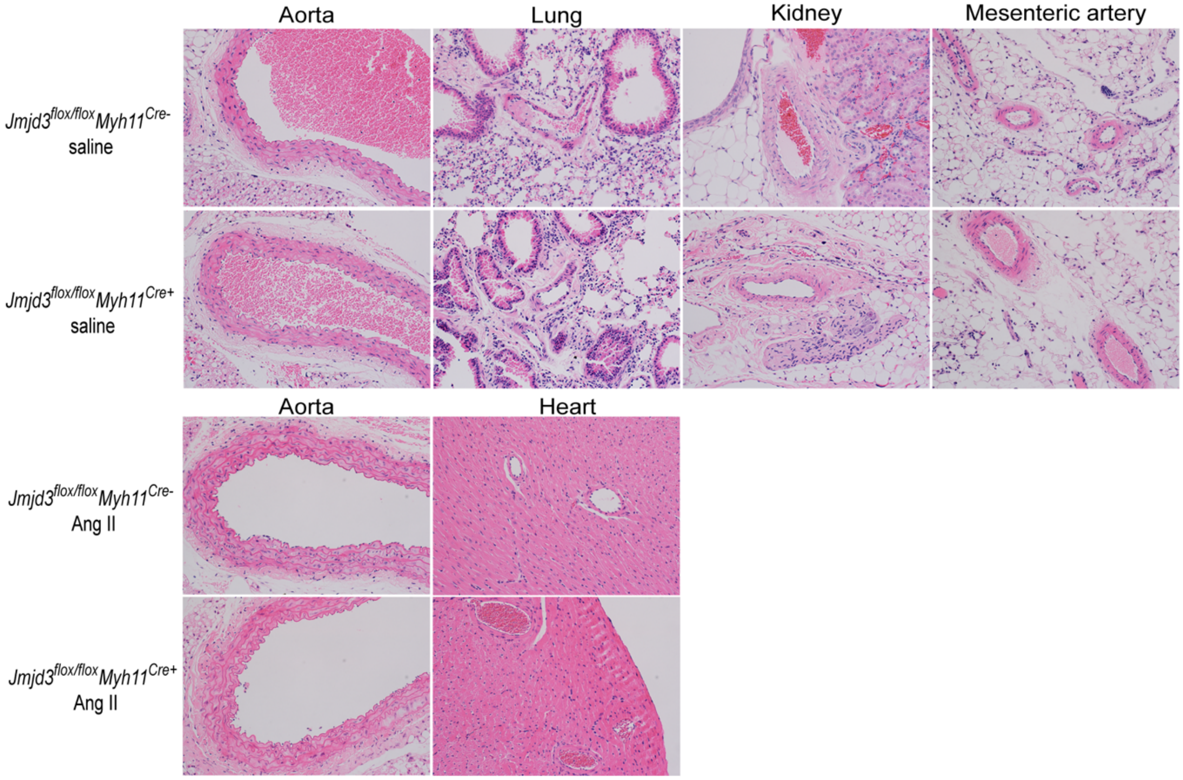

Supplemental Figure 6. H&E stained sections from multiple vascular containing tissues including aorta, lung, kidney, and mesenteric artery isolated from *Jmjd3*$^{flox/flox}$*Myhll*^{CreERT} under basal (saline-treated) conditions. Aorta and heart from *Jmjd3*$^{flox/flox}$*Myhll*^{CreERT} mice treated with Ang II are also depicted.
