# Supplementary material for: Epigenetic alteration of smooth muscle cells regulates endothelin-dependent blood pressure and hypertensive arterial remodeling
Source: J Clin Invest. 2025 Mar 27;135(11):e186146. doi: 10.1172/JCI186146 (PMC12126237; doi:10.1172/JCI186146)

Unedited gels and blots

Mangum et al JCI Resubmission

Figure 1F

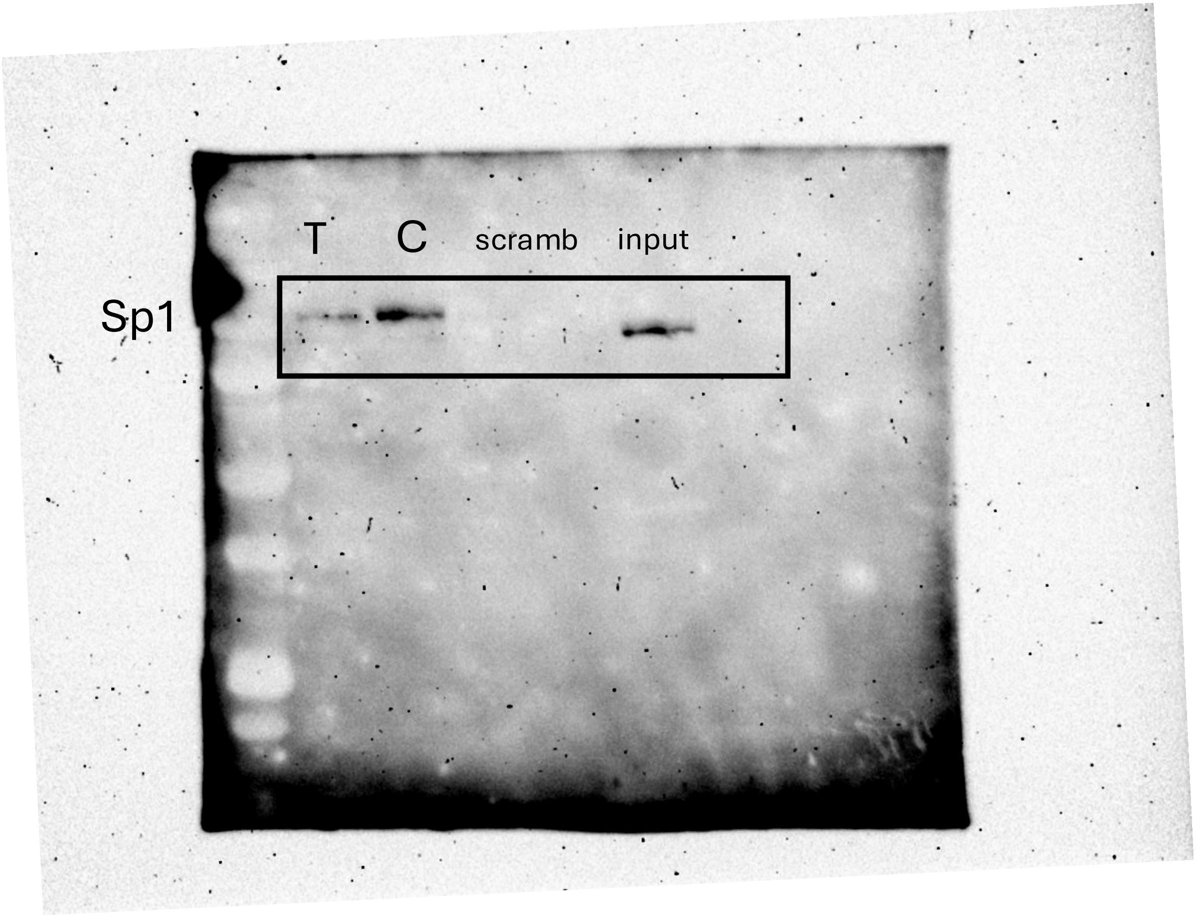

Figure 2E

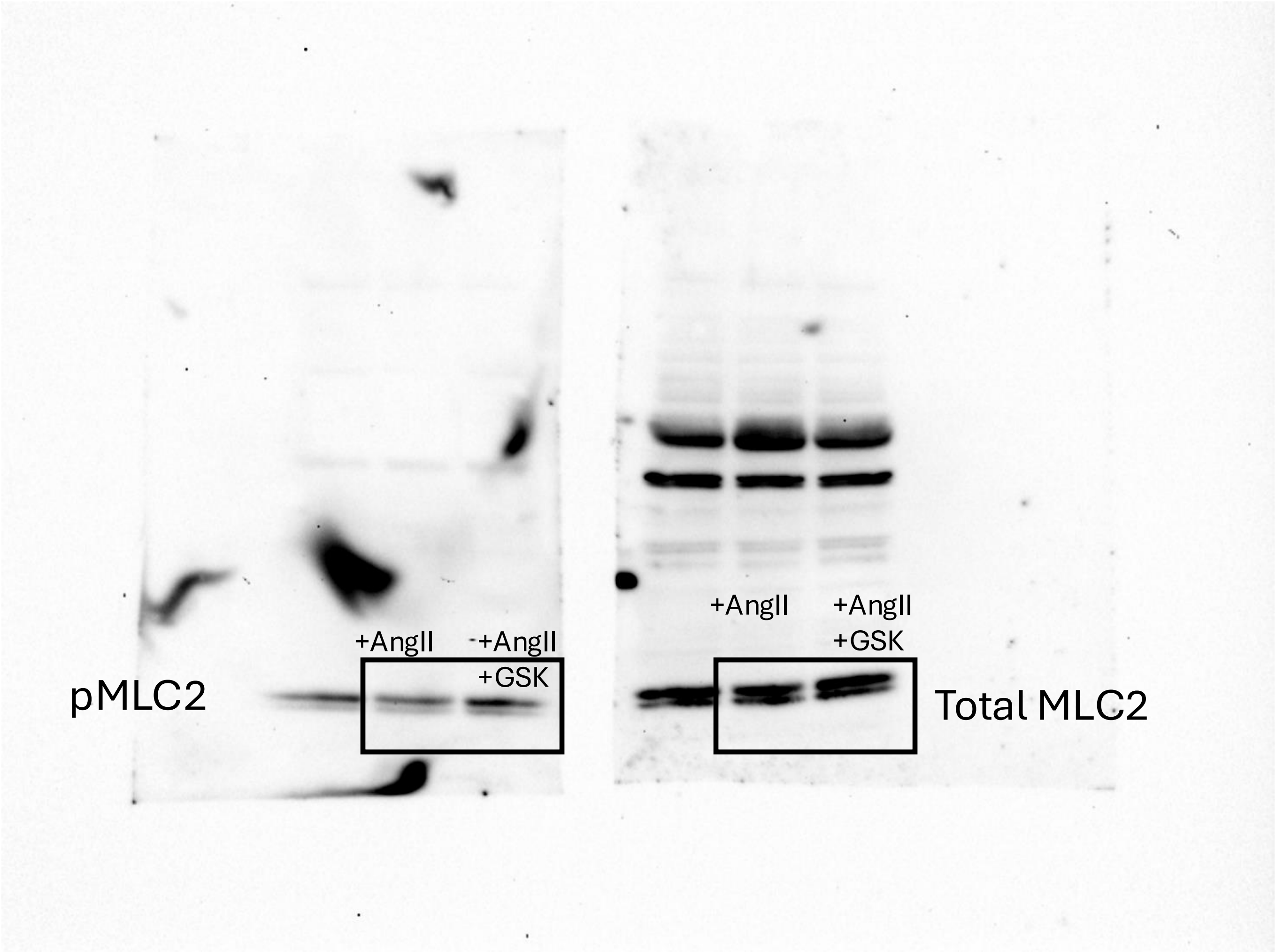

Figure 3D

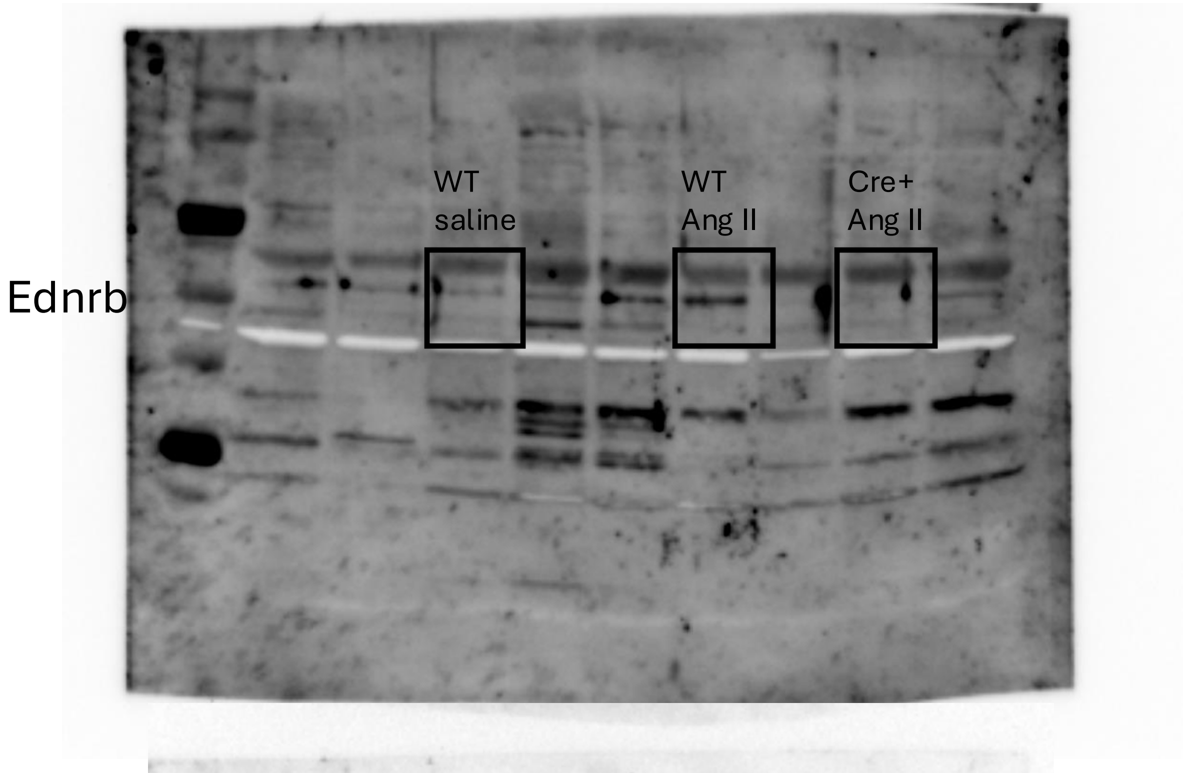

Ednrb
WT
saline
WT
Ang II
Cre+
Ang II

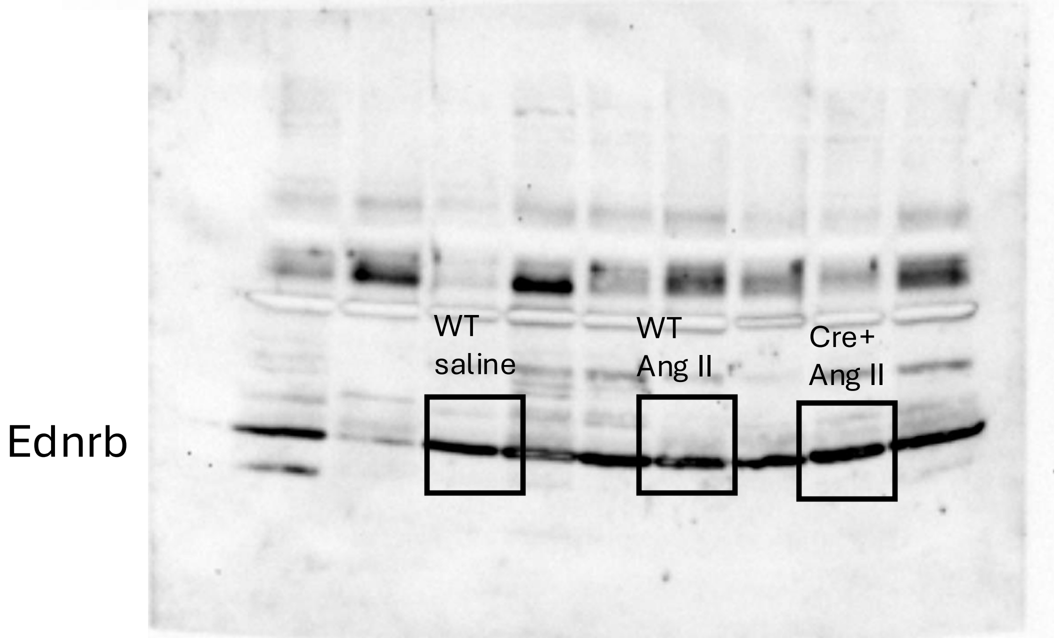

Ednrb
WT
saline
WT
Ang II
Cre+
Ang II

Figure 3O

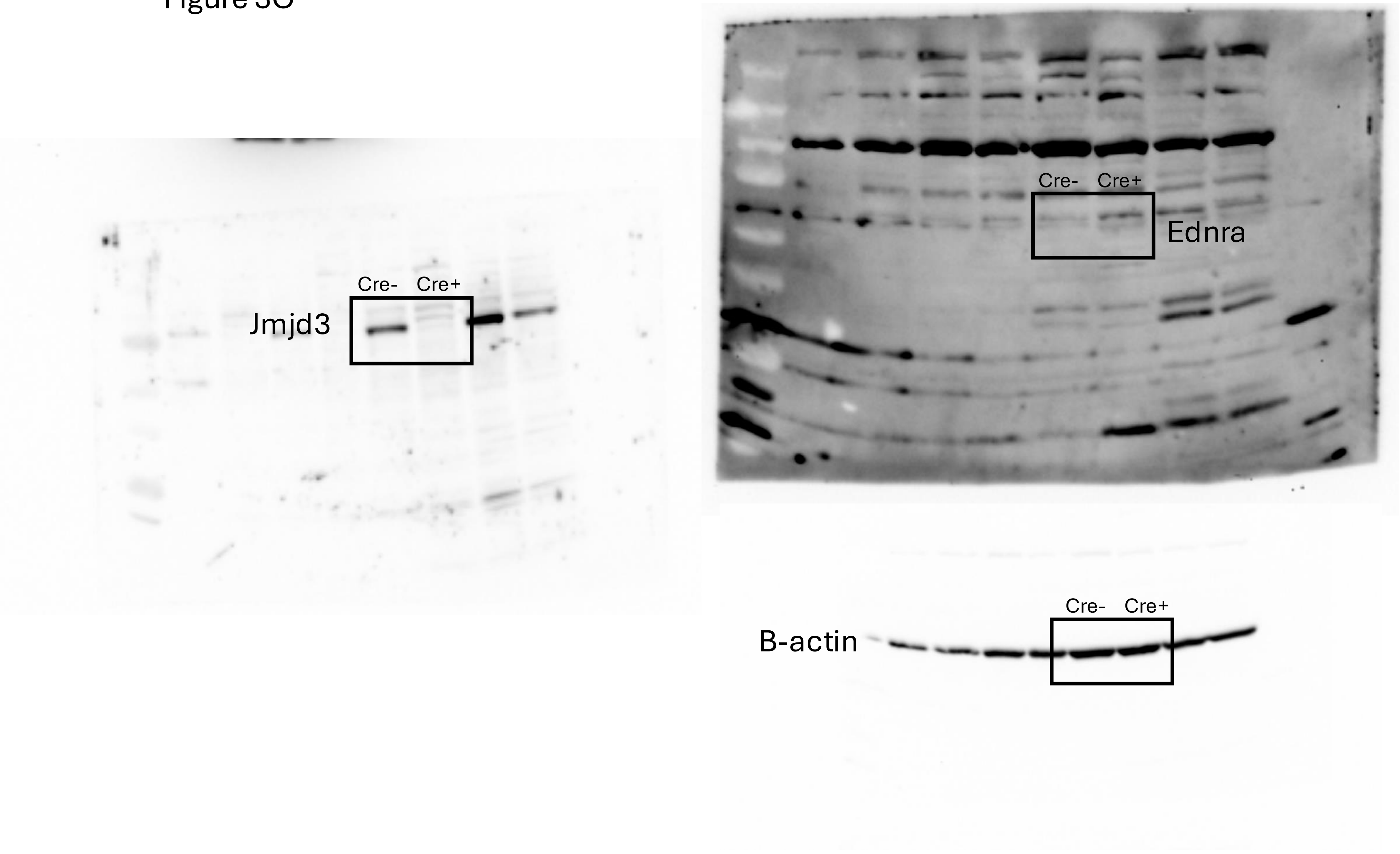

Figure 4E

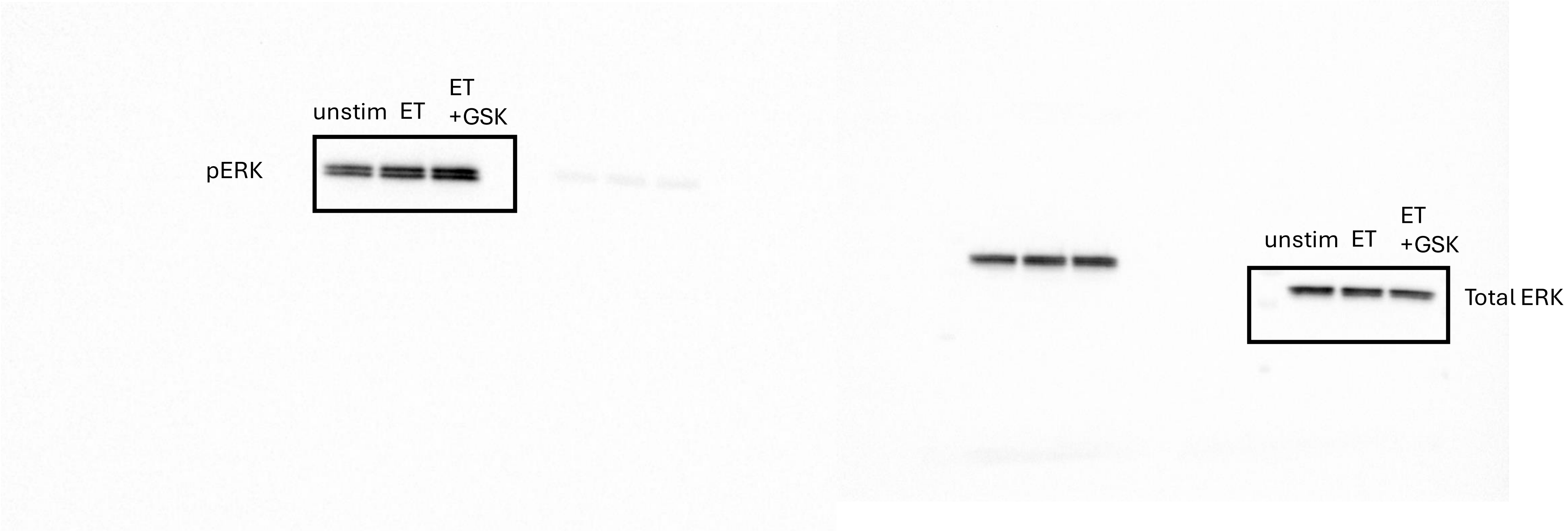

Figure 4F

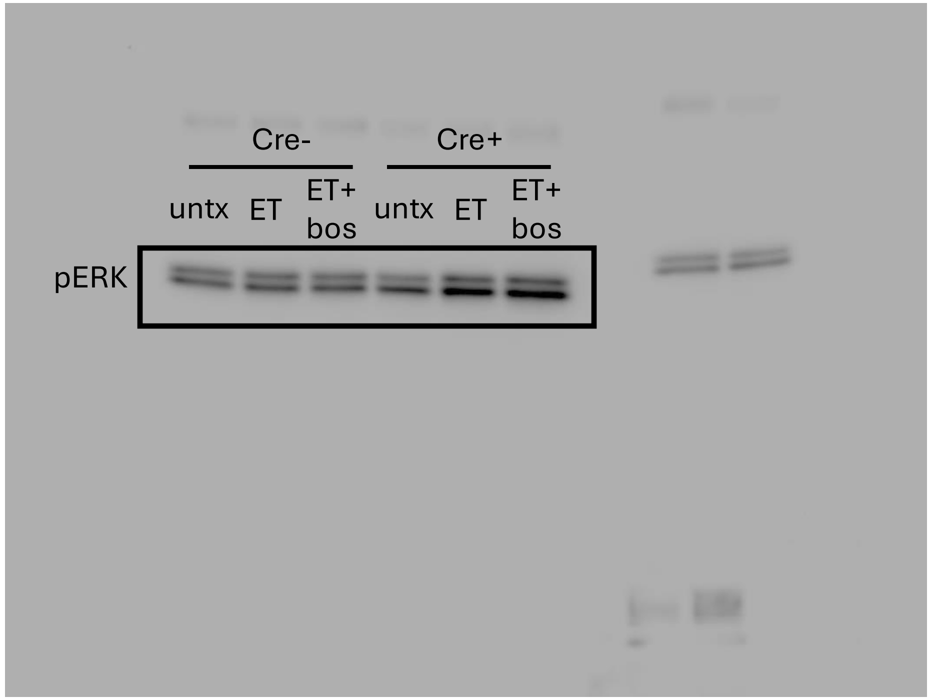
Cre-
Cre+
untx
ET
ET+ bos
untx
ET
ET+ bos
pERK

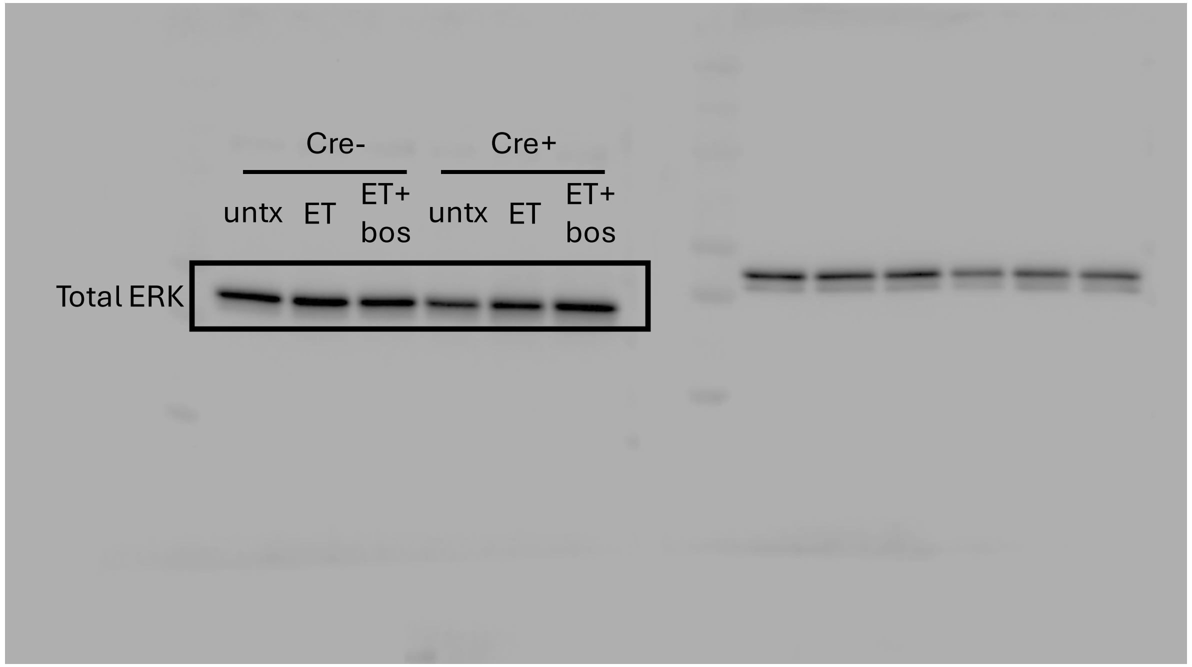
Cre-
Cre+
untx
ET
ET+ bos
untx
ET
ET+ bos
Total ERK

Figure 6D

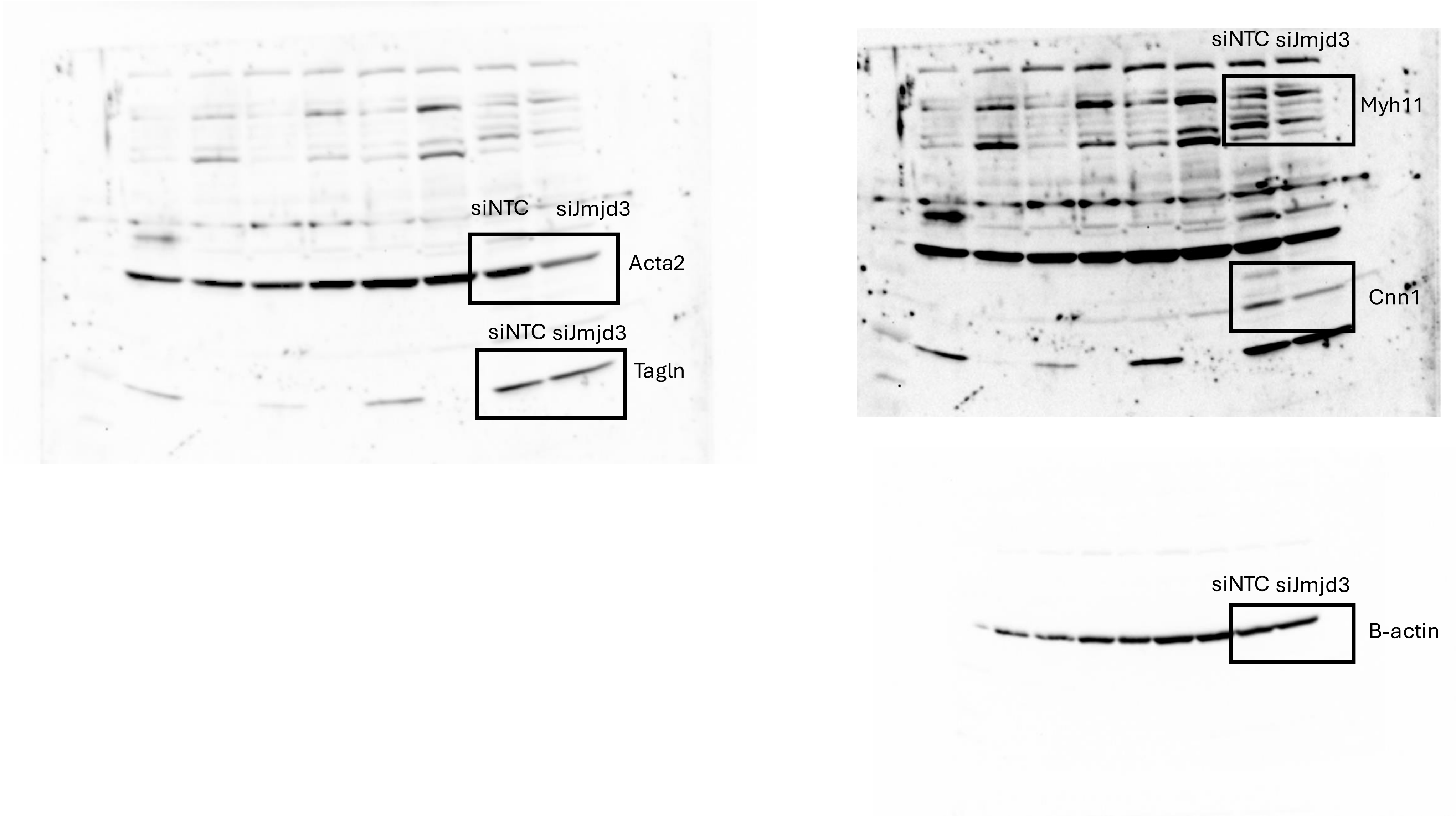
siNTC
siJmjd3
Acta2
siNTC siJmjd3
Tagln
siNTC siJmjd3
Myh11
Cnn1
siNTC siJmjd3
B-actin

Figure 7E

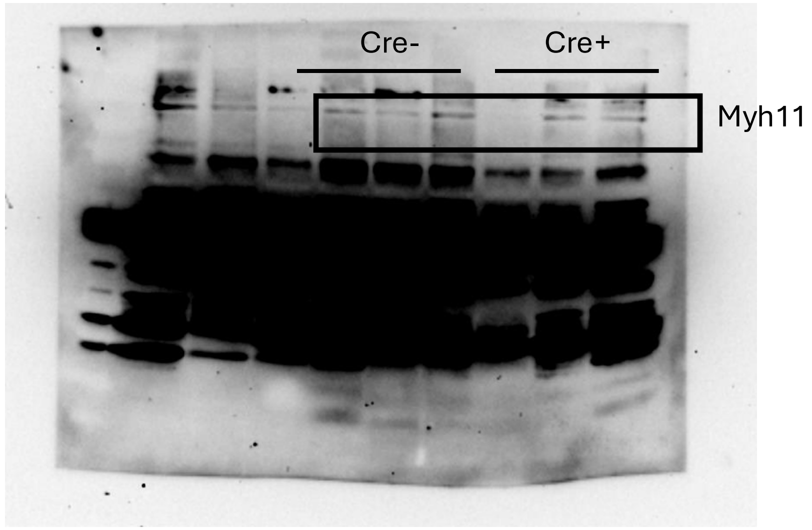

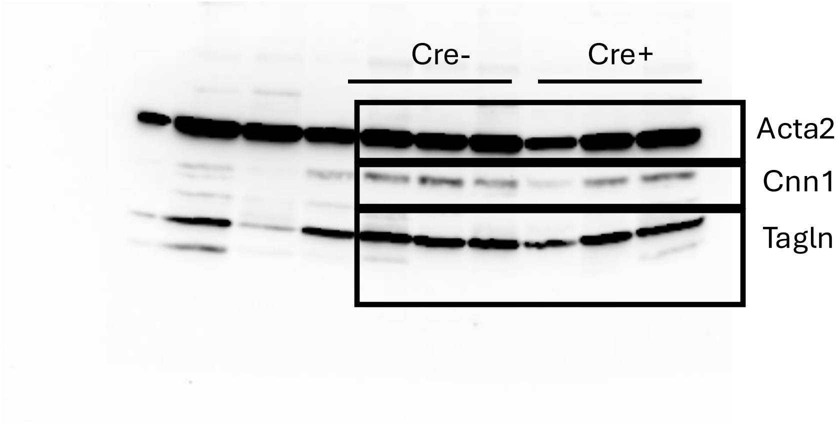

Figure 7G

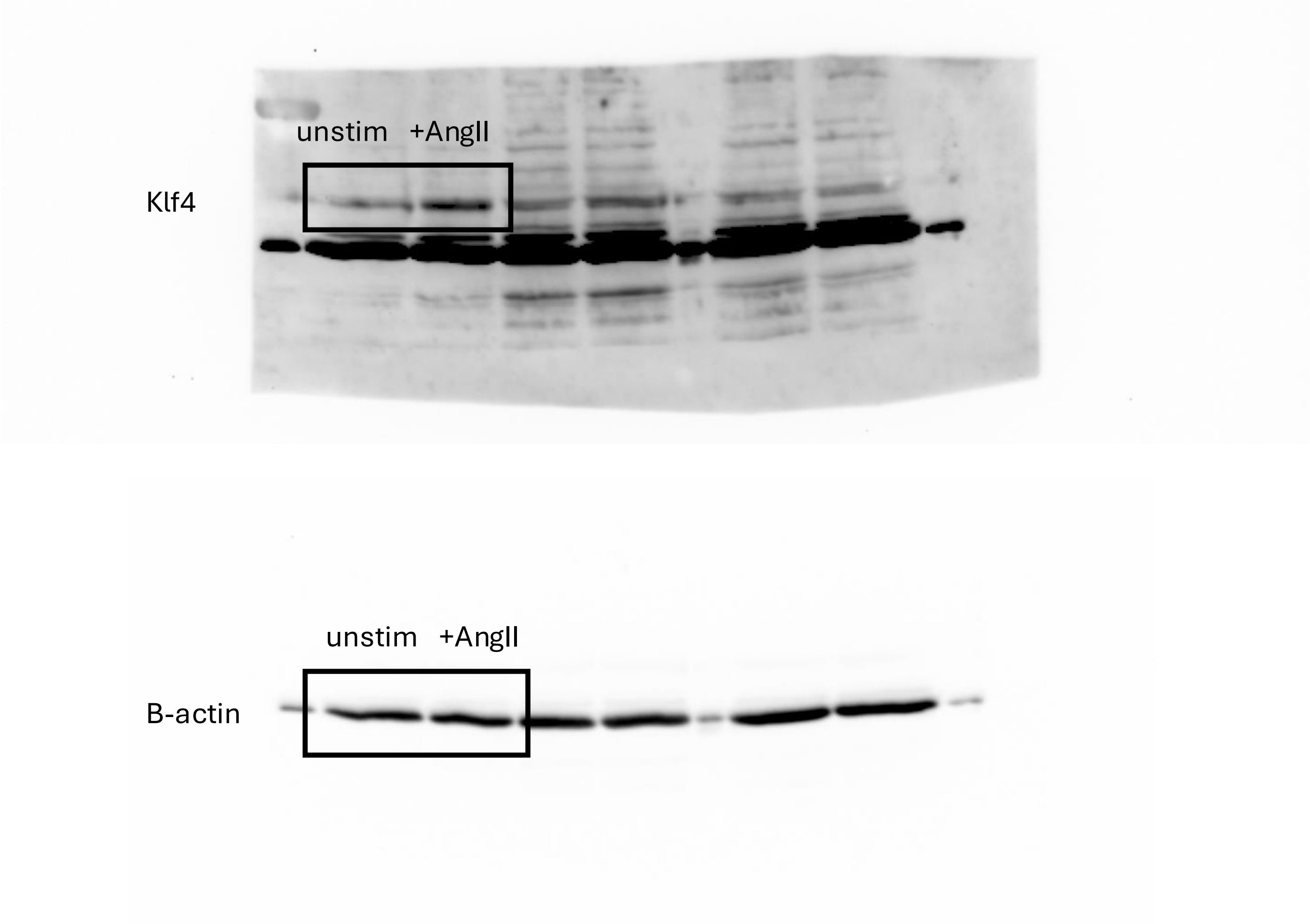

Supplemental Figure 1E

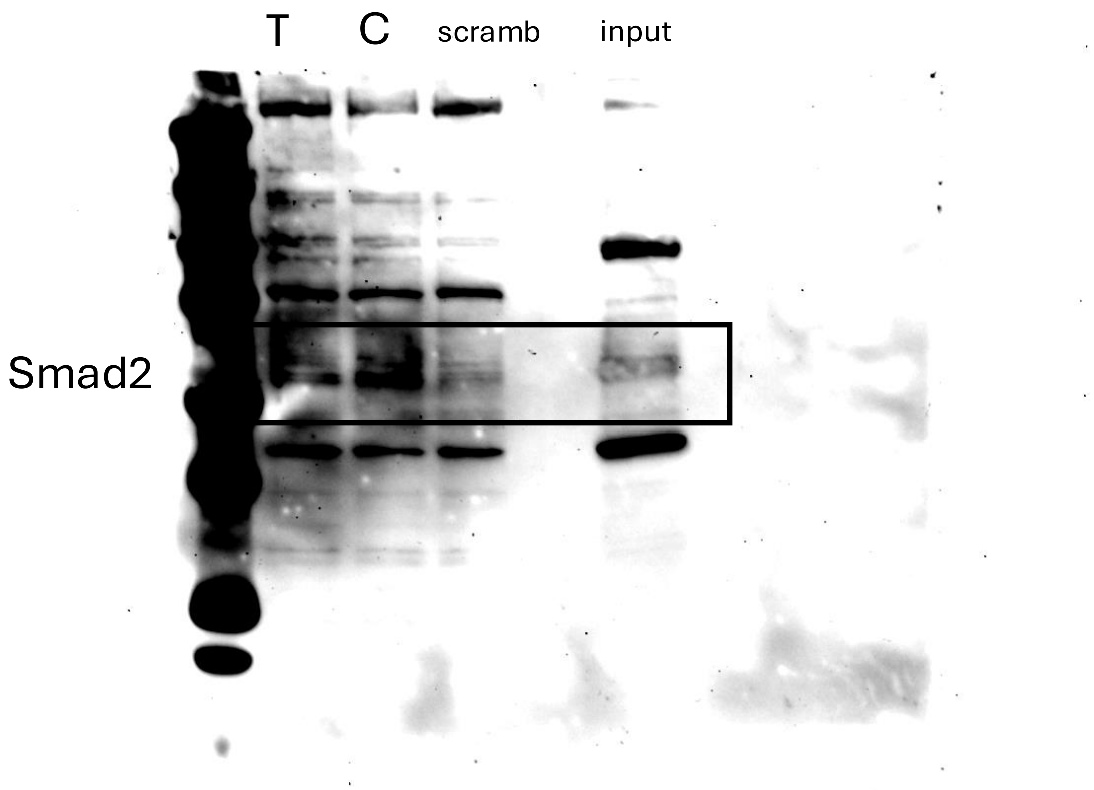

Supplemental Figure 3B

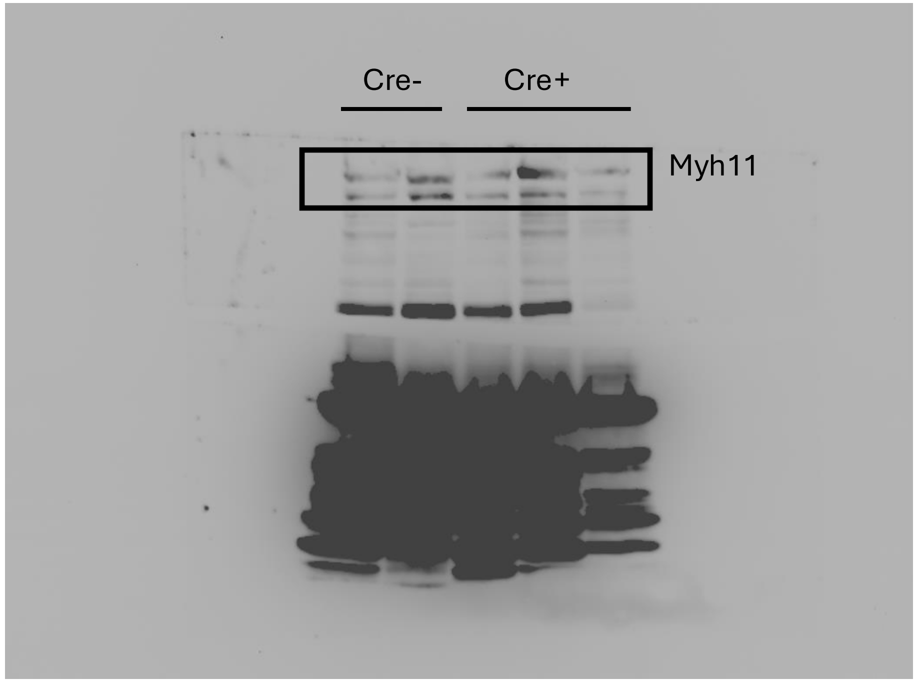

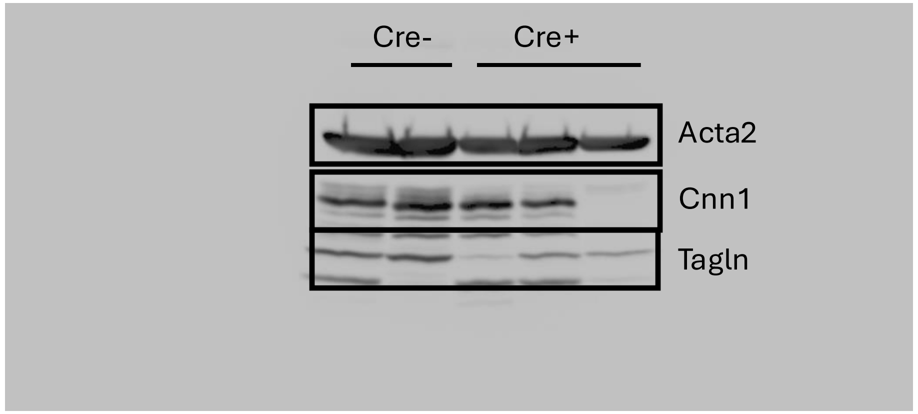

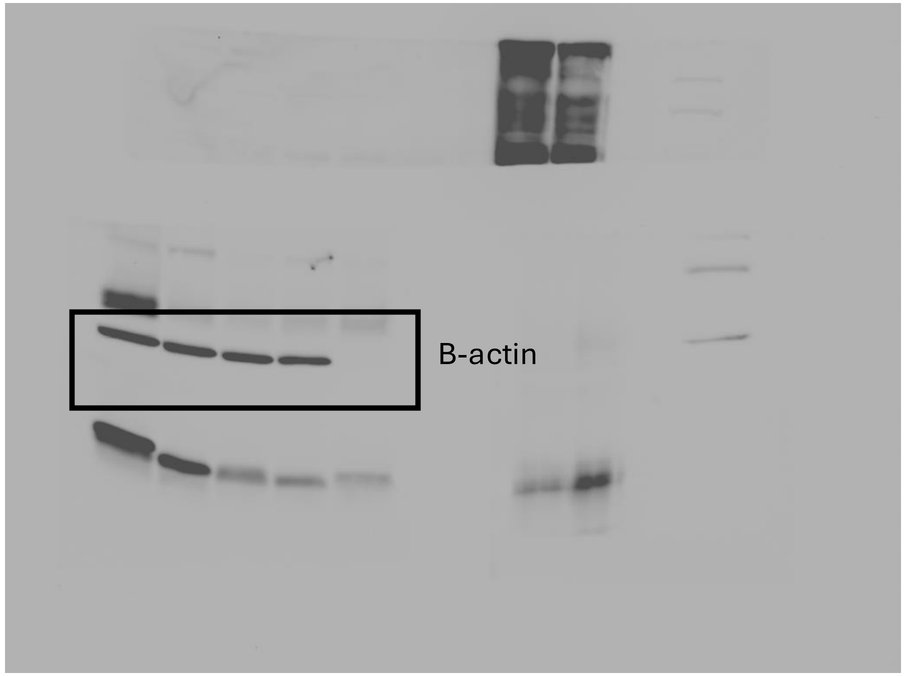

Supplement: Unedited blot and gel images [file jci-135-186146-s108.pdf]
