# Supplementary material for: Epigenetic alteration of smooth muscle cells regulates endothelin-dependent blood pressure and hypertensive arterial remodeling
Source: J Clin Invest. 2025 Mar 27;135(11):e186146. doi: 10.1172/JCI186146 (PMC12126237; doi:10.1172/JCI186146)
Supplement: Supplemental table 2 [file jci-135-186146-s110.pdf]

Antibodies

Antibody	Company (Cat.#)	Species	Dilution	Use*
Beta-actin	Cell signaling (3700S)	Mouse	1:1000	WB
Calponin	Cell signaling (DAL2T)	Rabbit	1:1000	WB
Endothelin receptor A	Thermofischer (PA3-065)	Rabbit	1:500	WB, IF
Endothelin receptor B	Thermofischer (PA3-066)	Rabbit	1:500	WB
ERK1/2	Cell signaling (9911T)	Rabbit	1:500	WB
Flag	Cell signalilng (14793S)	Rabbit	1:500	IF
H3K27me3	Active motif (39155)	Rabbit	1:1000	ChIP
JMJD3	Abcam (ab169197)	Rabbit	1:500	WB, ChIP
KLF4	Cell signaling (4038S)	Rabbit	1:500	WB
MCL2	Cell signaling (3672S)	Rabbit	1:500	WB
pERK1/2	Cell signaling (9911T)	Rabbit	1:500	WB, IF
pMCL2	Cell signaling (3674S)	Rabbit	1:500	WB
SM22	Santa Cruz (sc-53932)	Mouse	1:500	WB
SMAD2	Santa Cruz (sc-393312)	Mouse	1:500	WB
SMalpha-actin	Sigma (A5228)	Mouse	1:1000	WB
SM-MHC	Abcam (ab124679)	Rabbit	1:1000	WB
SP1	Cell signaling (9389S)	Rabbit	1:1000-500	WB, ChIP
SRF	Cell signaling (D71A9)	Rabbit	1:1000	ChIP

*WB, western blotting; IF, immunofluorescence; ChIP, chromatin immunoprecipitation
